# Discovery of Novel Quinazoline Derivatives as Potent Antitumor Agents

**DOI:** 10.3390/molecules27123906

**Published:** 2022-06-17

**Authors:** Zhenxi Niu, Shuli Ma, Lei Zhang, Qibing Liu, Shengnan Zhang

**Affiliations:** 1 Department of Pharmacy, Children’s Hospital Affiliated to Zhengzhou University, Henan Children’s Hospital, Zhengzhou Children’s Hospital, Zhengzhou 450018, China; zzuzhenxiniu@outlook.com (Z.N.); 13633860971@163.com (S.M.); 2 Department of Pharmacy, The First Affiliated Hospital of Hainan Medical University, Haikou 570100, China; zl0898@hainmc.edu.cn; 3 Martinos Center for Biomedical Imaging, Massachusetts General Hospital and Harvard Medical School, 149 Thirteenth Street, Suite 2301, Boston, MA 02129, USA

**Keywords:** quinazoline, antitumor, cell cycle, apoptosis, migration

## Abstract

In this work, we designed and synthesized a novel series of quinazoline derivatives **6**–**19** and then evaluated their broad-spectrum antitumor activity against MGC-803, MCF-7, PC-9, A549, and H1975, respectively. Most of them demonstrated low micromolar cytotoxicity towards five tested cell lines. In particular, compound **18** exhibited nanomolar level inhibitory activity against MGC-803 cells with an IC_50_ value of 0.85 μM, indicating approximately a 32-fold selectivity against GES-1 (IC_50_ = 26.75 μM). Further preclinical evaluation showed that compound **18** remarkably inhibited the migration of MGC-803 cells, induced cell cycle arrest at G2/M, and induced MGC-803 apoptosis, resulting in decreasing the expression of both Bcl-2 and Mcl-1, and up-regulating the expression of both Bax and cleaved PARP. No death or obvious pathological damage was observed in mice by acute toxicity assay. The in vivo antitumor evaluation suggested that compound **18** significantly decreased the average tumor volume and tumor weight without any effect on body weight, which is better than 5-Fu. Therefore, compound **18** can be used as a lead compound for the further development of antitumor drugs in the future.

## 1. Introduction

In 2020, there were approximately 19.3 million new cases of cancer and 10.0 million deaths from it [1]. Cancer cases will continue to increase from 19.3 to 28.4 million by 2040, which calls on scientists from the academic and pharmaceutical communities to actively look for prevention and treatment options [2]. To date, the top three options for cancer treatment are chemotherapy, radiotherapy, and surgery, of which chemotherapy drugs supply a unique method for systemic cancer therapy [3,4]. Although a lot of potent chemotherapy drugs have been used in the clinic, we still face two main challenges: (a) acquired resistance induced by long-term treatment with chemotherapy drugs; and (b) toxicity toward normal cells and tissues [5,6,7,8]. Therefore, the development of novel anticancer drugs to address drug resistance and avoid unwanted side effects are urgently needed.

**Figure 1:**
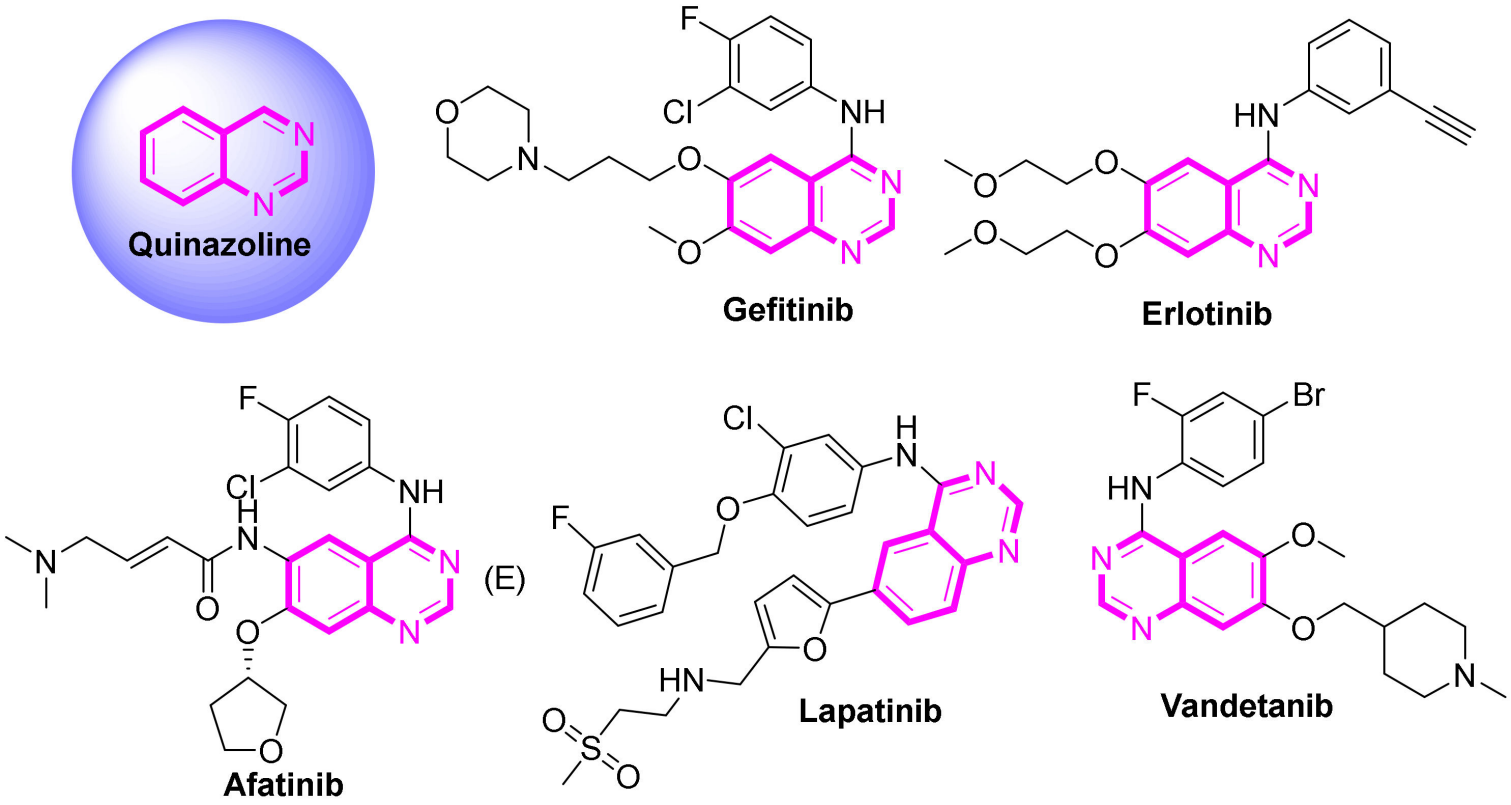
FDA approved quinazoline derivatives for cancer therapy.

Heterocyclic compounds are widely found in natural products and synthetic compounds, and most of them have been reported to possess various biological activities [9,10,11,12,13,14,15,16]. As a promising privileged nitrogen-containing heterocycle scaffold, quinazoline is formed by two fused six-member aromatic rings: pyrimidine and benzene. Many quinazoline derivatives have been found to possess extensive biological activities, including anticancer [17,18], antiviral [19], antimalarial [20], antimicrobial [21], anti-inflammatory [22,23,24], anticonvulsant [25,26], antioxidant [27], and antihypertensive [28], for example. Of note, several quinazoline derivatives, including gefitinib [29], erlotinib [30], vandetanib [31], afatinib [32], and lapatinib [33], have been approved by the United States Food and Drug Administration (USFDA) in the past for cancer therapy in the clinic because of their promising therapeutic efficacy against human cancers (Shown in Figure 1) [34]. With the emergence of drug resistance, the development of new small-molecule compounds that overcome drug resistance is still a problem facing us. Our research group has long been interested in the development of small-molecule anticancer drugs based on the privileged scaffold quinazoline. In this work, we designed and synthesized a novel series of quinazoline derivatives and then evaluated their anticancer activity.

## 2. Results and Discussion

### 2.1. Chemistry

**Scheme 1:**
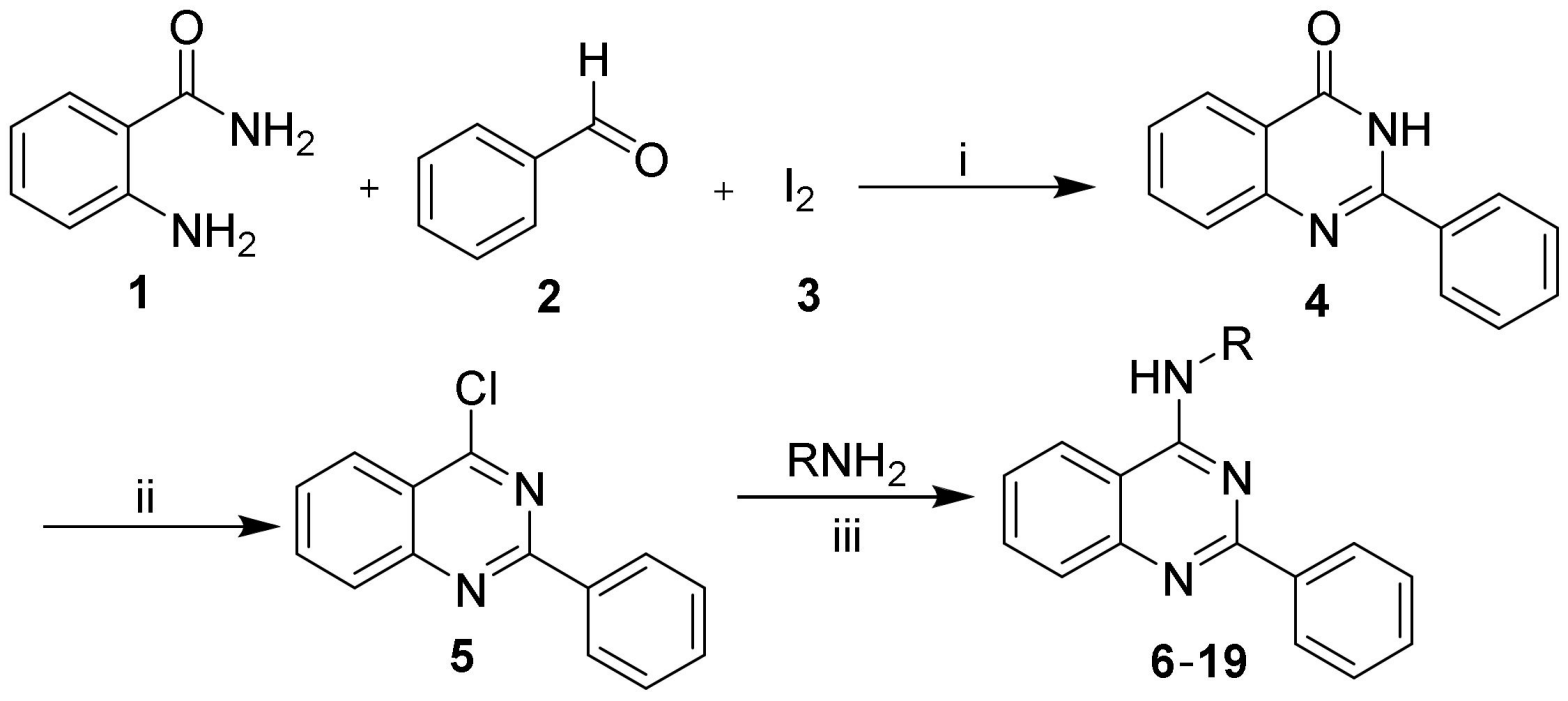
Synthesis of compounds **6**–**19**. Reagents and conditions: (i) EtOH, room temperature, 5 h; (ii) POCl_3_, 90 °C, 5 h; (iii) EtOH, room temperature, overnight.

The synthetic route for compounds **6**–**19** is shown in Scheme 1. To a solution of 2-aminobenzamide and benzaldehyde in ethanol was added iodine, affording 2-phenylquinazolin-4(3*H*)-one. 2-phenylquinazolin-4(3*H*)-one was then dissolved in phosphorus oxychloride under reflux to give the intermediate 4-chloro-2-phenylquinazoline. The target compounds **6**–**19** were finally achieved by reacting 4-chloro-2-phenylquinazoline with various amino derivatives in ethanol.

### 2.2. Biological Evaluation

In this work, we designed and synthesized a novel series of quinazoline derivatives **6**–**19**. Subsequently, we further evaluated their broad-spectrum antitumor activity against five different cancer cell lines, including MGC-803, MCF-7, PC9, A549, and H1975, respectively, and 5-Fu was selected as the positive control to ensure the reliability of our screening methods. The results are summarized in Table 1, which indicates that compounds **6**–**19** displayed weak to strong inhibitory activity toward the five selected cell lines. As shown in Table 1, compound **6** exhibited moderate inhibitory activity against MGC-803, MCF-7, PC9, A549, and H1975, among which compound **6** inhibited MGC-803 with an IC_50_ value of 6.23 μM. The incorporation of different halogen atoms (F, Cl, or Br) in the para position of the benzene ring in compound **6** afforded compound **7**, **8**, or **9**, respectively, and they exhibited increased antiproliferative activity against five cancer cell lines, among which the effect of compound **9** was better than compounds **7** and **8**. Of note, compound **9**, containing electron-withdrawing bromo atom at 4-position of phenyl, significantly inhibited MGC-803 with an IC_50_ value of 1.89 μM, while showing relatively weak inhibitory activity against the other four cell lines. However, replacing the bromo atom with strong electron-withdrawing nitro or trifluoromethyl group in compound **9** resulted in compounds **10** and **11** having decreased inhibitory activity against five cell lines. Further replacement of the bromo atom with a methyl or methoxy group in compound **9** afforded **12** and **13**, respectively, both of which showed comparable inhibitory activity with compound **6** but less than **9**. Introduction of the 3,4,5-trimethoxy group to the phenyl group of compound **6** yielded compound **14** with increased inhibitory activity, but still less than that of compound **9**. Similarly, the installation of the naphthalene group to replace the 3,4,5-trimethoxy group in compound **14** afforded compound **15**, which still exhibited dissatisfactory activity. 

We also explored the effect of the introduction of chalcone moiety on the inhibitory activity, affording compound **16**. Intriguingly, compound **16** exhibited remarkable improvement of inhibitory activity, relative to compound **6**, which showed comparable or better inhibitory activity when compared to **9**. Replacement of fluorine atom in **16** with chlorine or bromine led to compounds **17** and **18**, both of which possessed similar or better inhibitory activity against five cell lines relative to **16**. Compound **18** achieved particular nanomolar level inhibitory activity against MGC-803 with an IC_50_ value of 0.85 μM. However, the replacement of the phenyl group with a naphthalene ring in compound **18**, affording compound **19,** slightly decreased the inhibitory activity against the five cancer cell lines. 

Given the promising antiproliferative activity of **6**–**19**, we also explored their possible toxicity toward normal human gastric epithelial cell line (GES-1), including 5-Fu as a positive control. The results are summarized in Table 2. The data in Table 2 indicate that most of them exhibited relatively weak inhibition against GES-1, exhibiting significantly lower toxicity than 5-Fu. Intriguingly, the most potent compound, **18**, exhibited almost no inhibitory activity against GES-1, with an IC_50_ value of 26.75 μM, albeit with the fact that compound **18** significantly inhibited MGC-803 (IC_50_ = 0.85 μM), exhibiting remarkable selectivity (about 32-fold), which was then selected to further explore the underlying biological mechanism.

### 2.3. The Effect of Compound ***18*** on the Migration of MGC-803 Cells

**Figure 2:**
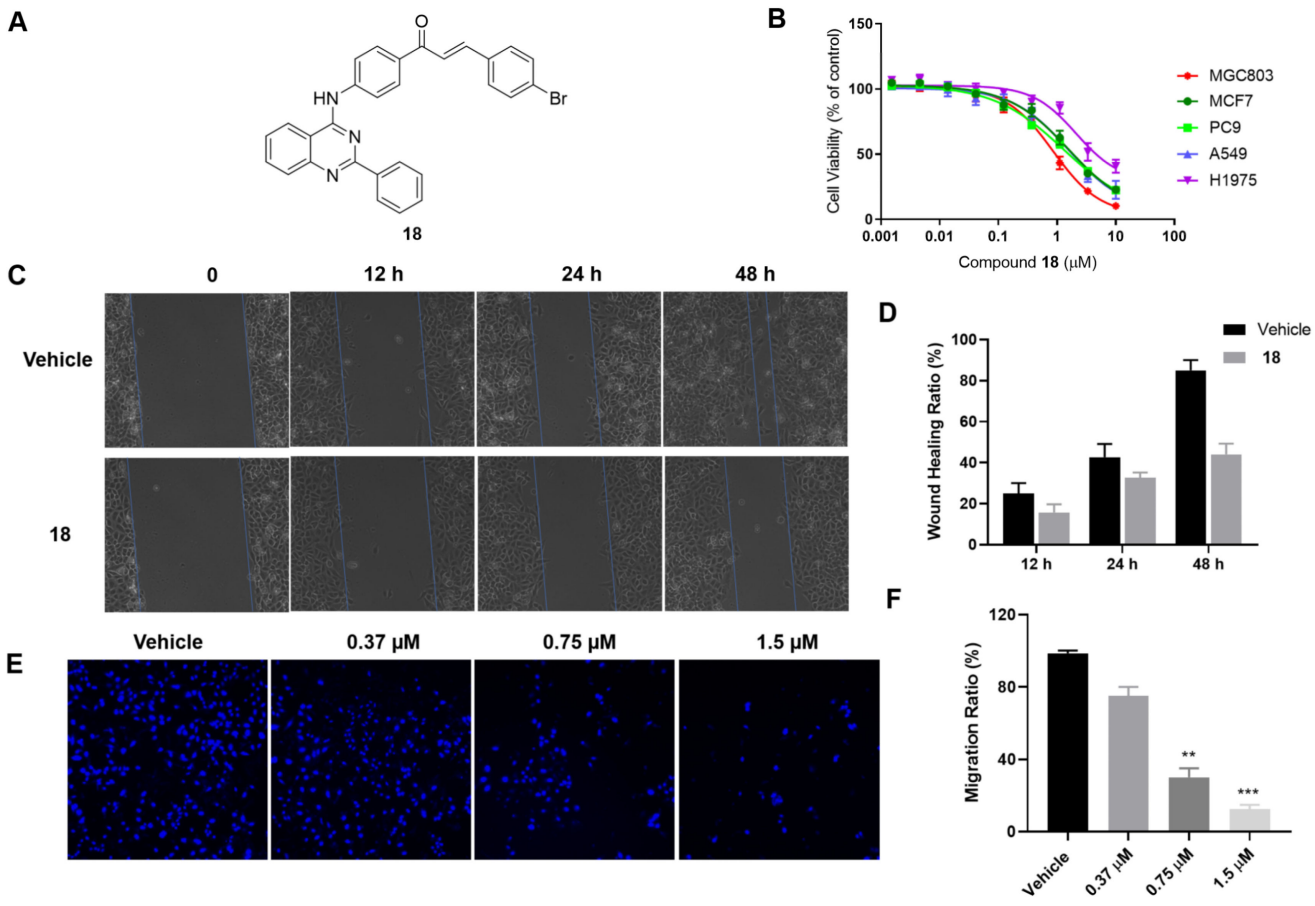
The effect of compound **18** on the migration of MGC-803 cells. (**A**) The structure of compound **18**; (**B**) The effect of **18** on the cell viability of selected cancer cells with different concentrations for 72 h by MTT method; (**C**,**D**) The effect of **18** at 1 µM on the migration of MGC-803 cells in a wound healing assay; (**E**,**F**) The effect of **18** on the migration of MGC-803 cells for 48 h in a transwell assay. ** *p* < 0.01, *** *p* < 0.001 compared to control group.

As shown in Figure 2A,B and Table 1, compound **18** dose-dependently inhibited the viability of the selected cells. It especially exhibited the strongest inhibitory activity against MGC-803 cells (IC_50_ = 0.85 μM). In addition to rampant cell proliferation, metastatic cancer is also characterized by cell migration, which was generally studied by using a wound healing assay and transwell assay. Therefore, we explored its effect on the migration of MGC-803 cells through a wound healing assay and a transwell assay. The results are summarized in Figure 2C–F, which indicate that compound **18** could effectively inhibit the migration of MGC-803 cells dose-dependently in both the wound healing assay and the transwell assay.

### 2.4. The Effect of Compound ***18*** on Apoptosis of MGC-803 Cells

**Figure 3:**
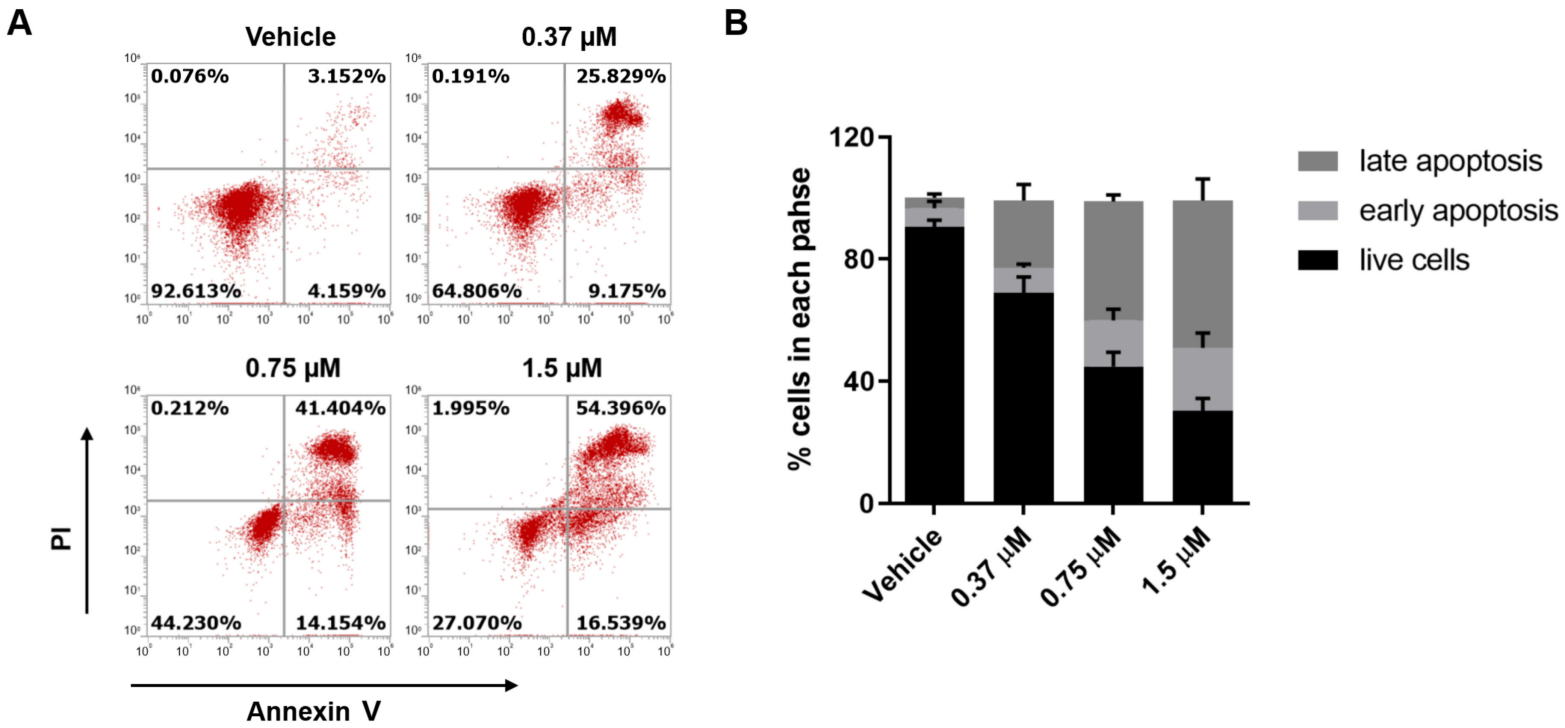
The effect of compound **18** on the apoptosis of MGC-803 cells. (**A**,**B**) MGC-803 cells were treated with compound **18** at the indicated concentrations for 48 h, which was then analyzed by using Annexin V-FITC/PI double staining through flow cytometry.

Given the potent inhibitory activity of compound **18** MGC-803 cells, a flow cytometric analysis was performed to quantitatively analyze the apoptosis-related change in MGC-803 cells. The results are summarized in Figure 3A,B. Treatment of MGC-803 cells with compound **18** at different concentrations for 48 h led to the dose-dependent increase of FITC-Annexin V/PI positive population. Especially at 1.5 μM, compound **18** induced significant apoptosis of MGC-803 cells with the value of apoptotic percentage up to 70.9% in both late and early apoptosis. These data indicated that compound **18** could induce both early and late apoptosis of MGC-803 cells in a dose-dependent manner. 

**Figure 4:**
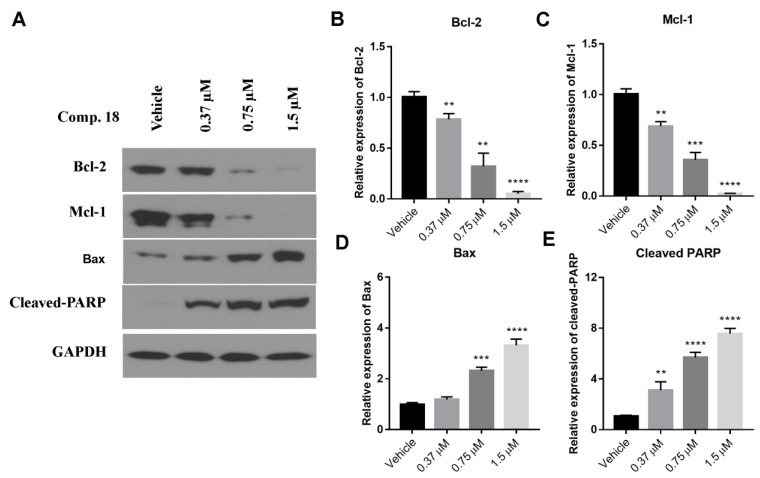
The effect of compound **18** on apoptosis-related proteins in MGC-803 cells. (**A**–**E**) Expression and quantitative analysis of cleaved-PARP, Mcl-1, Bcl-2, and Bax in MGC-803 cells treated with compound **18** at the different concentrations for 48 h. 0.001 < ** *p* < 0.01, *** *p* < 0.001, and **** *p* < 0.0001 compared to the control group.

The intrinsic pathway mediated by mitochondria is one of the main apoptotic pathways [21]. It has been reported that Bax increases mitochondrial stress, thereby inducing cytochrome C release, caspase-9 activation, and caspase activation pathway activation [22,23,24], while Bcl-2 plays an opposite role in response to a variety of apoptotic stimuli by blocking the release of mitochondrial cytochrome C [25]. To further explore the underlying mechanism of compound **18** on MGC-803 cells, we also explored the effect of compound **18** on the expression of apoptosis-associated proteins in MGC-803 cells. The results were summarized in Figure 4A–E. As shown in Figure 4A–D, compound **18** remarkably decreased the expression of anti-apoptotic proteins Bcl-2 and Mcl-1, while up-regulating the expression of the pro-apoptotic protein Bax in a dose-dependent manner. In addition, the extrinsic pathway mediated by death receptors is another major apoptotic pathway [27]. The caspase family, including caspase 9 and caspase 3, play an important role in cell apoptosis. Caspase 3 further cracks PARP and promotes cell disintegration, which is regarded as a marker of cell apoptosis [28]. Therefore, we also evaluated the effect of compound **18** on the expression of cleaved PARP in MGC-803 cells. As shown in Figure 4A,E, compound **18** dose-dependently induced the up-regulation of the expression of cleaved PARP. Our findings indicated that compound **18** mediated the apoptosis of MGC-803 cells through the activation of two major apoptotic pathways simultaneously.

### 2.5. The Effect of Compound ***18*** on Cell Cycle Distribution of MGC-803 Cells

**Figure 5:**
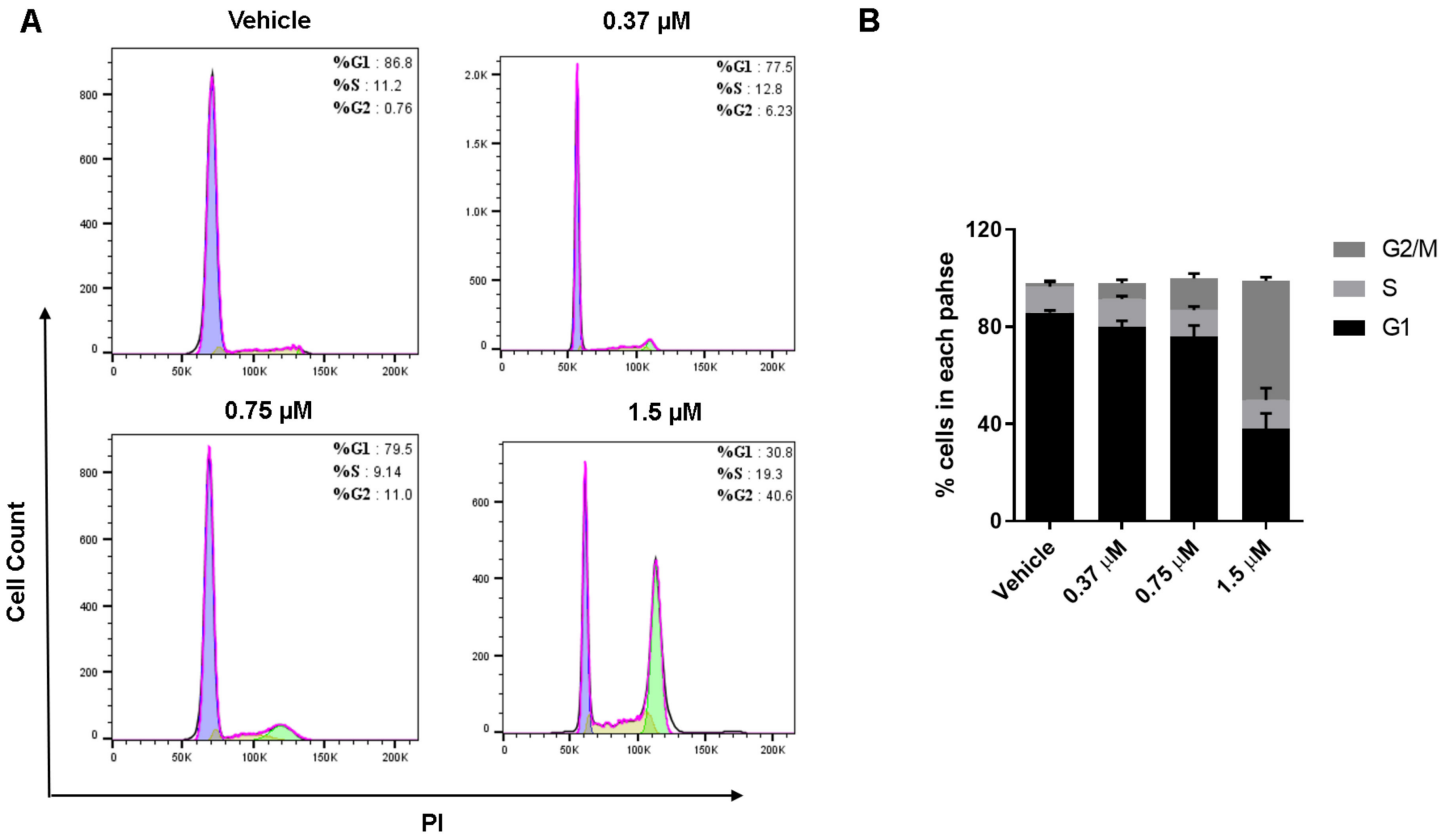
The effect of compound **18** on cell cycle distribution of MGC-803 cells. (**A**) MGC-803 cells were treated with compound **18** at the indicated concentrations for 48 h, which was then analyzed by flow cytometry; (**B**) Column chart of the cell cycle distribution of MGC-803 cells.

Loss of cell cycle control is the cause of the continued proliferation of cancer cells [18,19]. Therefore, we further evaluated the effect of compound **18** on the MGC-803 cell cycle. The results are summarized in Figure 5A,B. As depicted in Figure 5A,B, compound **18** significantly induced cell cycle arrest at G2/M, while it dose-dependently decreased the percentage of MGC-803 cells in the G1 phase.

### 2.6. Acute Toxicity Assay

**Figure 6:**
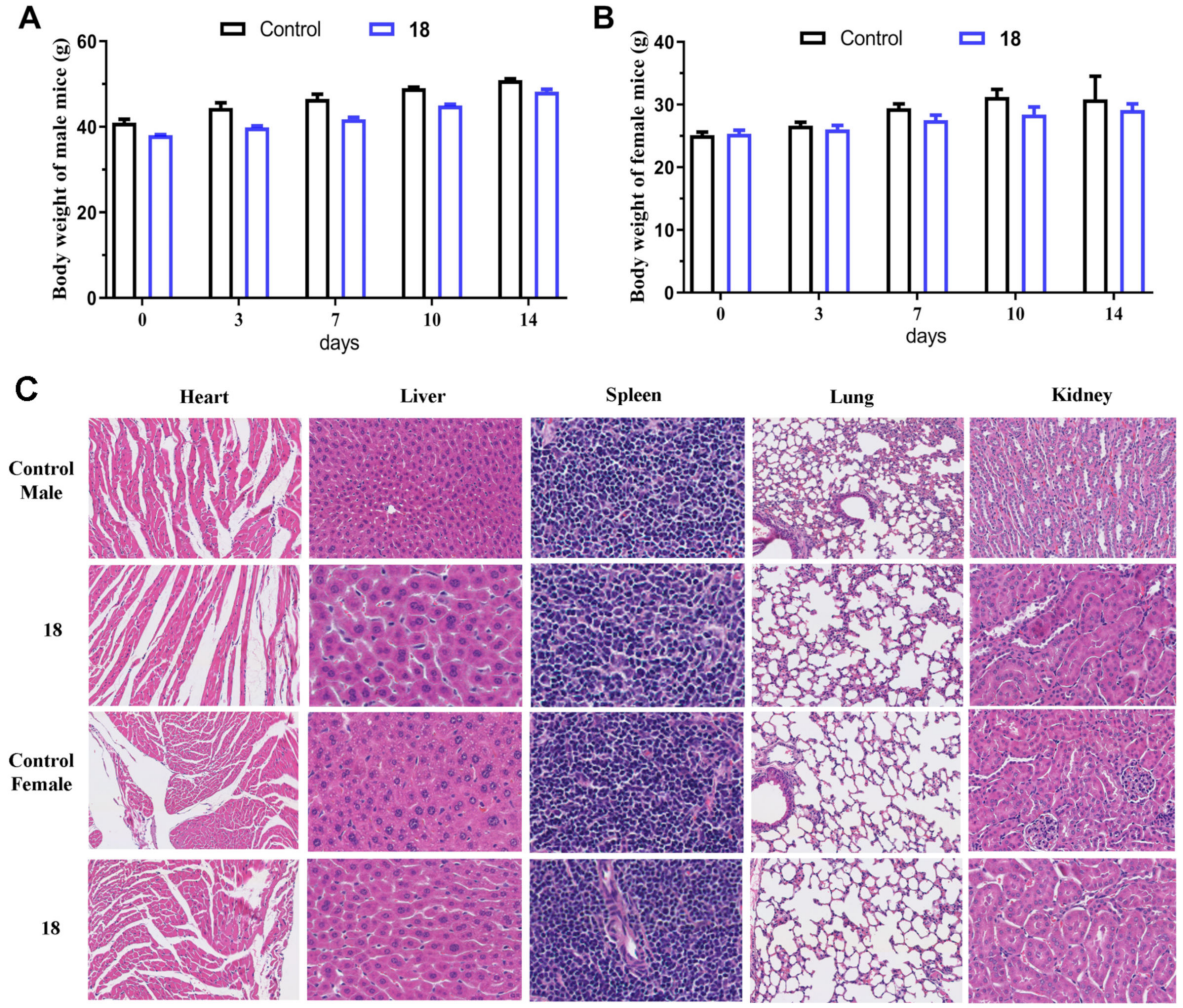
The safety evaluation of compound **18**. (**A**) Body weight of male mice-time; (**B**) Body weight of female mice-time; (**C**) The histological changes in mice were analyzed by HE staining (magnification: ×100).

Inspired by the fact that compound **18** possessed potent antiproliferative activity against MGC-803 cells in vitro and favorable drug-like properties, we further investigated the safety evaluation of compound **18** in healthy male and female mice through an acute toxicity assay. The results were summarized in Figure 6A,B. As shown in Figure 6A,B, intragastric administration of 1000 mg/kg compound **18** led to no death or obvious weight loss in healthy male and female mice compared to the control group. All mice in this acute toxicity assay grew normally, and their body weights increased gradually. In addition, the toxicity of **18** to the six important organs (brain, heart, liver, spleen, lung, and kidney) was also evaluated. and no marked pathological damage was observed by the hematoxylin−eosin (HE) staining assay (Figure 6C).

### 2.7. The Anti-Proliferative Effect of ***18*** on Gastric Cancer Xenograft Model

**Figure 7:**
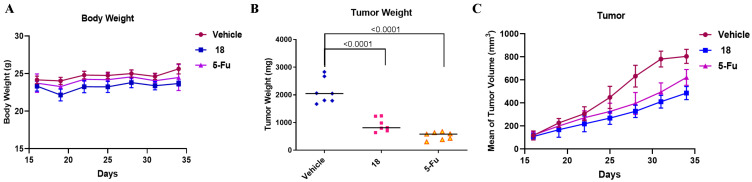
The in vivo anti-proliferative effect of **18** on the gastric cancer xenograft model. (**A**) The effect of compound **18** (25 mg/kg) on body weight, with 5-Fu (10 mg/kg) used as the control; (**B**) The effect of compound **18** (25 mg/kg) on the tumor weight, with 5-Fu (10 mg/kg) used as the control; (**C**) The effect of compound **18** (25 mg/kg) on the tumor volume, with 5-Fu (10 mg/kg) used as the control.

Given its promising antiproliferative activity against MGC-803 cells in vitro and the drug-like properties of compound **18**, the in vivo anti-tumor effect of compound **18** on xenograft model carrying MGC-803 cells was further explored through subcutaneous implantation, and 5-Fu was selected as the control. The body weight and tumor size of the mice were measured every three days. The results are summarized in Figure 7A–C. As shown in Figure 7A, compared with the control group, both compound **18** and 5-Fu did not cause significant changes in body weight, but decreased the average tumor volume and tumor weight (Figure 7B,C), exhibiting similar in vivo antitumor activity. 

## 3. Experimental Section

### 3.1. General Information

Reagents and solvents were commercially available. G-type silica gel (G60F-254) for thin-layer chromatography silica gel plate was used to monitor the chemical reaction, which was visualized by UV light (254 nm). Purification of target compounds by column chromatography used silica gel (200–300 mesh) obtained from the Qingdao Haiyang Chemical Co (Qingdao, China). An X-5 micromelting apparatus (Xiamen Ryder Scientific Instrument Co., Ltd., Xiamen, China) was used to measure melting points without correction. All of the NMR spectra were obtained by using a Bruker DPX 400 MHz spectrometer (Bruker, Karlsruhe, Germany), taking TMS as the internal standard in DMSO-*d_6_*. The value of δ ppm values was used to represent chemical shifts, relative to TMS. High-resolution mass spectra (HRMS) were achieved by Waters Micromass Q-T of a Micromass spectrometer (Waters, Milford, CT, USA) with electrospray ionization (ESI). 

### 3.2. Synthesis of Compounds ***4*** and ***5***

Compounds **4** and **5** were obtained according to the previously reported literature [35].

### 3.3. Synthesis of Compounds ***6***–***19***

4-Chloro-2-phenylquinazoline (240.7 mg, 1 mmol) reacted with aniline (93.13 mg, 1 mmol) in ethanol under room temperature overnight, which was monitored by thin-layer chromatography. Upon completion of the reaction, purification of the mixture was by column chromatography, finally resulting in compound **6** (217.5 mg, 0.73 mmol). Compounds **7**–**19** were also obtained according to the same synthetic method as compound **6**. The ^1^H- and ^13^C-NMR and HRMS spectra of compounds **6**–**19** can be found in the Supplementary Materials.

*N*,2-Diphenylquinazolin-4-amine (**6**), white solid, yield: 73%. m.p.: 171–175 °C. ^1^H-NMR (400 MHz, DMSO-*d_6_*) δ 11.68 (s, 1H), 8.97 (d, *J* = 8.2 Hz, 1H), 8.39 (d, *J* = 7.7 Hz, 3H), 8.09 (t, *J* = 7.7 Hz, 1H), 7.84 (dd, *J* = 18.8, 7.8 Hz, 3H), 7.70 (t, *J* = 7.2 Hz, 1H), 7.63 (t, *J* = 7.5 Hz, 2H), 7.55 (t, *J* = 7.7 Hz, 2H), 7.36 (t, *J* = 7.3 Hz, 1H). ^13^C-NMR (100 MHz, DMSO-*d_6_*) δ 158.95, 157.15, 136.86, 135.83, 133.24, 131.65, 129.15, 128.96, 128.69, 128.06, 126.41, 124.52, 120.67, 112.68. HRMS (ESI): *m/z* calcd for C_20_H_16_N_3_ (M + H)^+^, 298.1344; found, 298.1334.

*N*-(4-Fluorophenyl)-2-phenylquinazolin-4-amine (**7**), white solid, yield: 74%. m.p.: 181–186 °C. ^1^H-NMR (400 MHz, DMSO-*d_6_*) δ 11.82 (s, 1H), 8.99 (d, *J* = 8.3 Hz, 1H), 8.39 (t, *J* = 8.6 Hz, 3H), 8.09 (t, *J* = 7.7 Hz, 1H), 7.91–7.79 (m, 3H), 7.71 (t, *J* = 7.2 Hz, 1H), 7.63 (t, *J* = 7.5 Hz, 2H), 7.39 (t, *J* = 8.8 Hz, 2H). ^13^C-NMR (100 MHz, DMSO-*d_6_*) δ 158.94, 157.22, 135.94, 133.24, 131.68, 130.16, 129.22, 129.01, 128.62, 128.08, 126.12, 124.64, 120.85, 112.76. HRMS (ESI): *m/z* calcd for C_20_H_15_FN_3_ (M + H)^+^, 316.1250; found, 316.1240.

*N*-(4-Chlorophenyl)-2-phenylquinazolin-4-amine (**8**), white solid, yield: 56%. m.p.: 203–206 °C. ^1^H-NMR (400 MHz, DMSO-*d_6_*) δ 11.77 (s, 1H), 9.00 (d, *J* = 8.3 Hz, 1H), 8.40 (d, *J* = 7.3 Hz, 3H), 8.09 (t, *J* = 7.7 Hz, 1H), 7.92 (d, *J* = 8.7 Hz, 2H), 7.82 (t, *J* = 7.7 Hz, 1H), 7.71 (t, *J* = 7.2 Hz, 1H), 7.63 (dd, *J* = 18.7, 8.2 Hz, 4H). ^13^C-NMR (100 MHz, DMSO-*d_6_*) δ 158.92, 157.20, 135.93, 133.22, 131.67, 130.14, 129.20, 128.99, 128.61, 128.06, 126.10, 124.62, 120.83, 112.75. HRMS (ESI): *m/z* calcd for C_20_H_15_ClN_3_ (M + H)^+^, 332.0955; found, 332.0946.

*N*-(4-Bromophenyl)-2-phenylquinazolin-4-amine (**9**), white solid, yield: 60%. m.p.: 187–193 °C. ^1^H-NMR (400 MHz, DMSO-*d_6_*) δ 11.47 (s, 1H), 8.88 (d, *J* = 8.3 Hz, 1H), 8.37 (d, *J* = 7.3 Hz, 2H), 8.27 (d, *J* = 8.4 Hz, 1H), 8.09 (t, *J* = 7.7 Hz, 1H), 7.84 (dd, *J* = 17.8, 8.2 Hz, 3H), 7.78–7.61 (m, 5H). ^13^C-NMR (100 MHz, DMSO-*d_6_*) δ 158.95, 157.22, 135.95, 133.25, 131.69, 130.16, 129.23, 129.01, 128.63, 128.09, 126.12, 124.65, 120.86, 112.77. HRMS (ESI): *m/z* calcd for C_20_H_15_BrN_3_ (M + H)^+^, 376.0449; found, 376.0442.

*N*-(4-Nitrophenyl)-2-phenylquinazolin-4-amine (**10**), white solid, yield: 46%. m.p.: 199–203 °C. ^1^H-NMR (400 MHz, DMSO-*d_6_*) δ 11.13 (s, 1H), 8.80 (d, *J* = 8.2 Hz, 1H), 8.46–8.39 (m, 4H), 8.29 (d, *J* = 9.3 Hz, 2H), 8.13 (d, *J* = 8.3 Hz, 1H), 8.06 (t, *J* = 7.4 Hz, 1H), 7.80 (t, *J* = 7.2 Hz, 1H), 7.63 (dd, *J* = 7.9, 4.5 Hz, 3H). ^13^C-NMR (100 MHz, DMSO-*d_6_*) δ 158.43, 158.18, 144.70, 143.07, 135.06, 131.95, 128.88, 128.63, 127.46, 124.57, 123.88, 122.52, 113.60. HRMS (ESI): *m/z* calcd for C_20_H_15_N_4_O_2_ (M + H)^+^, 343.1195; found, 343.1186.

2-Phenyl-*N*-(4-(trifluoromethyl)phenyl)quinazolin-4-amine (**11**), white solid, yield: 83%. m.p.: 190–195 °C. ^1^H-NMR (400 MHz, DMSO-*d_6_*) δ 11.63 (s, 1H), 8.98 (d, *J* = 8.3 Hz, 1H), 8.41 (d, *J* = 7.3 Hz, 2H), 8.32 (d, *J* = 8.3 Hz, 1H), 8.18 (d, *J* = 8.4 Hz, 2H), 8.10 (t, *J* = 7.7 Hz, 1H), 7.91 (d, *J* = 8.5 Hz, 2H), 7.83 (t, *J* = 7.6 Hz, 1H), 7.67 (m, 3H). ^13^C-NMR (100 MHz, DMSO-*d_6_*) δ 159.04, 157.60, 141.10, 135.77, 132.93, 129.11, 128.98, 128.18, 127.98, 125.84, 125.80, 125.57, 124.51, 124.12, 122.87, 121.80, 113.02. HRMS (ESI): *m/z* calcd for C_21_H_15_F_3_N_3_ (M + H)^+^, 366.1218; found, 366.1208.

2-Phenyl-*N*-(*p*-tolyl)quinazolin-4-amine (**12**), white solid, yield: 66%. m.p.: 169–172 °C. ^1^H-NMR (400 MHz, DMSO-*d_6_*) δ 11.45 (s, 1H), 8.87 (d, *J* = 8.2 Hz, 1H), 8.35 (d, *J* = 7.3 Hz, 2H), 8.28 (d, *J* = 8.3 Hz, 1H), 8.08 (t, *J* = 7.7 Hz, 1H), 7.82 (t, *J* = 7.7 Hz, 1H), 7.72 (dd, *J* = 13.9, 7.7 Hz, 3H), 7.64 (t, *J* = 7.4 Hz, 2H), 7.35 (d, *J* = 8.2 Hz, 2H), 2.39 (s, 3H). ^13^C-NMR (100 MHz, DMSO-*d_6_*) δ 158.71, 157.47, 135.50, 134.56, 134.43, 132.87, 131.68, 129.13, 128.92, 127.79, 125.12, 124.20, 124.02, 112.82, 20.68. HRMS (ESI): *m/z* calcd for C_21_H_18_N_3_ (M + H)^+^, 312.1501; found, 312.1491.

*N*-(4-Methoxyphenyl)-2-phenylquinazolin-4-amine (**13**), white solid, yield: 72%. m.p.: 196–201 °C. ^1^H-NMR (400 MHz, DMSO-*d_6_*) δ 11.66 (s, 1H), 8.94 (d, *J* = 8.2 Hz, 1H), 8.39 (d, *J* = 7.5 Hz, 3H), 8.08 (t, *J* = 7.7 Hz, 1H), 7.87–7.57 (m, 6H), 7.10 (d, *J* = 8.9 Hz, 2H), 3.83 (s, 3H). ^13^C-NMR (100 MHz, DMSO-*d_6_*) δ 158.64, 157.51, 157.05, 135.74, 133.28, 129.52, 129.17, 128.97, 128.01, 125.92, 124.50, 120.27, 113.81, 112.59, 55.34. HRMS (ESI): *m/z* calcd for C_21_H_18_N_3_O (M + H)^+^, 328.1450; found, 328.1440.

2-Phenyl-*N*-(3,4,5-trimethoxyphenyl)quinazolin-4-amine (**14**), white solid, yield: 70%. m.p.: 185–189 °C. ^1^H-NMR (400 MHz, DMSO-*d_6_*) δ 11.30 (s, 1H), 8.93 (d, *J* = 8.3 Hz, 1H), 8.46 (d, *J* = 7.2 Hz, 2H), 8.31 (d, *J* = 8.3 Hz, 1H), 8.08 (t, *J* = 7.7 Hz, 1H), 7.81 (t, *J* = 7.6 Hz, 1H), 7.68 (m, 3H), 7.41 (s, 2H), 3.84 (s, 6H), 3.73 (s, 3H). ^13^C-NMR (100 MHz, DMSO-*d_6_*) δ 158.40, 157.51, 152.54, 135.35, 133.31, 132.82, 128.94, 128.83, 127.72, 124.20, 112.94, 101.55, 99.97, 60.22, 55.96. HRMS (ESI): *m/z* calcd for C_23_H_22_N_3_O_3_ (M + H)^+^, 388.1661; found, 388.1650.

*N*-(Naphthalen-1-yl)-2-phenylquinazolin-4-amine (**15**), white solid, yield: 66%. m.p.: 204–208 °C. ^1^H-NMR (400 MHz, DMSO-*d_6_*) δ 12.17 (s, 1H), 9.08 (d, *J* = 8.0 Hz, 1H), 8.48 (d, *J* = 8.3 Hz, 1H), 8.17–8.00 (m, 6H), 7.89 (t, *J* = 7.6 Hz, 1H), 7.78 (d, *J* = 7.1 Hz, 1H), 7.71 (t, *J* = 7.7 Hz, 1H), 7.58 (m, 3H), 7.45 (t, *J* = 7.7 Hz, 2H). ^13^C-NMR (100 MHz, DMSO-*d_6_*) δ 160.80, 157.04, 135.95, 133.79, 133.14, 132.82, 131.35, 129.17, 128.94, 128.72, 128.30, 128.16, 128.06, 126.52, 126.44, 125.59, 124.93, 124.75, 123.43, 120.56, 112.54. HRMS (ESI): *m/z* calcd for C_24_H_18_N_3_ (M + H)^+^, 348.1501; found, 348.1492.

(*E*)-3-(4-Fluorophenyl)-1-(4-((2-phenylquinazolin-4-yl)amino)phenyl)prop-2-en-1-one (**16**), yellow solid, yield: 85%. m.p.: 252–256 °C. ^1^H-NMR (400 MHz, DMSO-*d_6_*) δ 11.41 (s, 1H), 8.93 (d, *J* = 8.2 Hz, 1H), 8.45 (d, *J* = 6.9 Hz, 2H), 8.36 (d, *J* = 8.6 Hz, 2H), 8.27 (d, *J* = 8.1 Hz, 1H), 8.20 (d, *J* = 8.5 Hz, 2H), 8.11–7.99 (m, 4H), 7.81 (dd, *J* = 16.8, 11.7 Hz, 2H), 7.67 (dd, *J* = 14.9, 7.3 Hz, 3H), 7.33 (t, *J* = 8.7 Hz, 2H). ^13^C-NMR (100 MHz, DMSO-*d_6_*) δ 187.66, 164.63, 162.16, 158.69, 157.83, 142.49, 142.17, 135.37, 132.47, 131.44, 131.28, 131.19, 129.45, 128.93, 128.86, 127.70, 124.21, 122.88, 121.87, 116.02, 115.80, 113.32. HRMS (ESI): *m/z* calcd for C_29_H_21_FN_3_O (M + H)^+^, 446.1669; found, 446.1658.

(*E*)-3-(4-Chlorophenyl)-1-(4-((2-phenylquinazolin-4-yl)amino)phenyl)prop-2-en-1-one (**17**), yellow solid, yield: 79%. m.p.: 263–267 °C. ^1^H-NMR (400 MHz, DMSO-*d_6_*) δ 11.24 (s, 1H), 8.89 (d, *J* = 8.1 Hz, 1H), 8.45 (d, *J* = 6.8 Hz, 2H), 8.36 (d, *J* = 8.4 Hz, 2H), 8.21 (d, *J* = 8.3 Hz, 3H), 8.11–8.03 (m, 2H), 7.97 (d, *J* = 8.3 Hz, 2H), 7.80 (dd, *J* = 18.0, 11.6 Hz, 2H), 7.66 (d, *J* = 7.0 Hz, 3H), 7.55 (d, *J* = 8.2 Hz, 2H). ^13^C-NMR (100 MHz, DMSO-*d_6_*) δ 187.59, 185.84, 158.55, 157.99, 142.19, 135.04, 133.73, 130.57, 130.33, 129.53, 128.93, 128.89, 128.73, 127.51, 124.50, 124.06, 123.47, 122.71, 116.68, 113.43. HRMS (ESI): *m/z* calcd for C_29_H_21_ClN_3_O (M + H)^+^, 462.1373; found, 462.1365.

(*E*)-3-(4-Bromophenyl)-1-(4-((2-phenylquinazolin-4-yl)amino)phenyl)prop-2-en-1-one (**18**), yellow solid, yield: 57%. m.p.: 247–251 °C. ^1^H-NMR (400 MHz, DMSO-*d_6_*) δ 11.30 (s, 1H), 8.91 (d, *J* = 8.3 Hz, 1H), 8.46 (d, *J* = 6.5 Hz, 2H), 8.38 (d, *J* = 8.6 Hz, 2H), 8.27–8.18 (m, 4H), 8.15–8.05 (m, 2H), 7.90 (d, *J* = 7.7 Hz, 1H), 7.84–7.72 (m, 2H), 7.66 (d, *J* = 7.4 Hz, 4H), 7.44 (t, *J* = 7.8 Hz, 1H). ^13^C-NMR (100 MHz, DMSO-*d_6_*) δ 187.57, 158.64, 157.88, 142.51, 141.94, 137.25, 135.30, 133.63, 132.99, 132.41, 130.93, 130.74, 129.58, 128.92, 128.82, 128.30, 127.62, 124.19, 123.42, 122.78, 122.38, 113.36. HRMS (ESI): *m/z* calcd for C_29_H_21_BrN_3_O (M + H)^+^, 506.0868; found, 506.0860.

(*E*)-3-(Naphthalen-2-yl)-1-(4-((2-phenylquinazolin-4-yl)amino)phenyl)prop-2-en-1-one (**19**), yellow solid, yield: 69%. m.p.: 281–286 °C. ^1^H-NMR (400 MHz, DMSO-*d_6_*) δ 11.43 (s, 1H), 8.93 (d, *J* = 8.1 Hz, 1H), 8.48–8.34 (m, 5H), 8.27 (d, *J* = 8.2 Hz, 1H), 8.18 (dd, *J* = 18.7, 8.8 Hz, 4H), 8.08 (t, *J* = 7.5 Hz, 1H), 7.97 (dd, *J* = 21.8, 12.2 Hz, 4H), 7.82 (t, *J* = 7.4 Hz, 1H), 7.67 (d, *J* = 7.5 Hz, 3H), 7.62–7.55 (m, 2H). ^13^C-NMR (100 MHz, DMSO-*d_6_*) δ 187.66, 158.63, 157.92, 143.74, 142.37, 135.28, 133.91, 132.95, 132.40, 130.64, 129.50, 128.94, 128.79, 128.51, 128.44, 127.71, 127.42, 126.78, 124.50, 124.46, 124.10, 122.77, 122.23, 113.36. HRMS (ESI): *m/z* calcd for C_33_H_24_N_3_O (M + H)^+^, 478.1919; found, 478.1910.

### 3.4. Cell Culture

Human gastric carcinoma cell line MGC-803, human breast adenocarcinoma cell line MCF-7, human lung carcinoma cell lines (PC-9, A549, and H1975), and human immortalized gastric mucosa epithelial cell line GES-1 were purchased from the Cell Bank of Shanghai Institute of Biochemistry and Cell Biology (Shanghai, China), and all of them were cultured in RPMI-1640 and DMEM complete medium (Solarbio, Beijing, China) at 37 °C in a 5% CO_2_ humidified atmosphere. 

### 3.5. MTT Assay

Briefly, cells were incubated with different concentrations of the test compound for different hours in each experiment for 72 h, followed by adding 20 μL MTT solution to each well, which was then incubated for another 4 h. An enzyme-linked immunosorbent assay reader (BioTek, South Plainfield, VT, USA) was used to measure absorbance values at 490 nm, and SPSS20 software was used to calculate the cell viability and IC_50_ values.

### 3.6. Migration Assay

For the wound healing assay, MGC-803 cells were seeded into 6-well plates, reaching the density of 90%, followed by being scraped off using a sterile yellow pipette tip and photographed. Subsequently, cells were treated with compound **18** at 1 µM for 12 h, 24 h, and 48 h, respectively, and then photographed again each time.

For the transwell assay, 600 µL of fresh medium was added to 20% FBS to the substrate of the 24-well plates. Then, 2 × 10^4^ MGC-803 cells were seeded into each well, followed by incubation with 0.37 µM, 0.75 µM, and 1.5 µM compound **18** in the upper chambers for 48 h, which was then stained by DAPI solution and photographed.

### 3.7. Cell Apoptosis

Cells were plated in 6-well plates and incubated with 0.37 µM, 0.75 µM, and 1.5 µM compound **18** for 48 h, followed by being harvested and stained by PI and Annexin V according to manufacturer’s protocol. Finally, flow cytometry (BD Bioscience, Franklin Lake, NJ, USA) was applied to detect apoptotic cells.

### 3.8. Cell Cycle

MGC-803 cells were seeded into 6-well plates at a concentration of 4 × 10^5^ cells per well, followed by being incubated with 0.37 µM, 0.75 µM, and 1.5 µM compound **18** for 48 h, which were subsequently harvested and stained in 500 µL Propidium Iodide solution (PI 1 mg/mL, RNase A 10 mg/mL, Solarbio, Beijing, China) at room temperature without light for 30 min. Finally, flow cytometry (BD Bioscience, Franklin Lake, NJ, USA) was used to detect and analyze staining cells.

### 3.9. Western Blot

Cells were incubated with 0.37 µM, 0.75 µM, and 1.5 µM compound **18** for 48 h and then collected with trypsin and total proteins and extracted in a lysis buffer. A BCA protein assay kit (Solarbio, Beijing, China) was used to confirm the concentration of the total proteins. These samples were detected by the standard protocol of western blot as described before [36].

### 3.10. Acute Toxicity Assay

The solution of compound **18** was prepared in 80% PEG400 solution. Eight ICR mice (four male/four female, 24–28 g, aged five to six weeks) were obtained from the Beijing Vital River Laboratory Animal Technology Co., Ltd. (Beijing, China). All animal treatments were performed under the guidelines of the National Institutes of Health Guide for the Care and Use of Laboratory Animals using an approved animal protocol by the Zhengzhou University Committee on Animal Care. Male and female mice (24–45 g) were divided randomly into four groups: a male control group (*n* = 2), a female control group (*n* = 2), a male group (*n* = 2), and a female group (*n* = 2). All mice were deprived of feed for 12 h. 1 g/kg compound **18** was administered intragastrically in the experiment group of mice, while the mice in the control group received the same volume of vehicle solution on the first day, followed by monitoring abnormal behaviors, body weight, and the death of these mice every day during the following two weeks. 

### 3.11. In Vivo Anti-Tumor Activity

Male mice (23–25 g, aged 5–6 weeks) were obtained from Beijing Vital River Laboratory Animal Technology Co., Ltd. (Beijing, China). All animal treatments were performed under the guidelines of the National Institutes of Health Guide for the Care and Use of Laboratory Animals using an approved animal protocol by the Zhengzhou University Committee on Animal Care (2020CHEM-ZZU-086). 1.0 × 10^7^ MGC-803 cells were implanted in the right flank of mice subcutaneously. After 16 days, the tumor volume reached 100 mm^3^, followed by being randomly divided into three groups (*n* = 7): solvent group, 5-Fu (10 mg/kg) group, and compound **18** (25 mg/kg) group. An intravenous injection of 5-Fu (10 mg/kg) was administered into the corresponding mice group every three days, while the mice in compound **18** group received intragastric administration of 25 mg/kg compound **18** per day for 21 days. Tumor diameter and body weight were measured every two days, followed by the euthanization of the mice after 34 days, after which tumor weights were.

## 4. Conclusions

In summary, a novel series of quinazoline derivatives were designed, synthesized, and evaluated for their broad-spectrum anticancer activity against MGC-803, MCF-7, PC-9, A549, and H1975. Most of them demonstrated low micromolar cytotoxicity toward five tested cell lines. Compound **18**, in particular, had the most antiproliferative effects against MGC-803 cells with an IC_50_ value of 0.85 μM. Studies conducted with compound **18** in MGC-803 cells demonstrated that it effectively inhibited migration, arrested the cell cycle at G2/M, and induced apoptosis. Compound **18** remarkably decreased the expression of Bcl-2 and Mcl-1, while upregulating the expression of Bax and cleaved PARP. In addition, a single dose of compound **18** did not cause significant acute toxicity and pathological damage. Further in vivo antitumor evaluation suggested that intragastric administration of 25 mg/kg compound **18** remarkably decreased the average tumor volume and tumor weight with no significant changes of body weight in mice. Overall, our study provides that compound **18** can be seen as a starting point for further research.

## Figures and Tables

**Table 1:** Antiproliferative activity of compounds **6**–**19** against selected cell lines.

Compound	R	IC_50_ (μM)				
		MGC-803	MCF-7	PC-9	A549	H1975
**6**	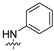	6.23 ± 0.12	12.62 ± 0.16	15.81 ± 0.52	10.31 ± 0.98	20.08 ± 1.62
**7**	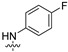	4.15 ± 1.16	8.75 ± 1.76	10.62 ± 2.16	4.82 ± 0.99	12.58 ± 2.89
**8**	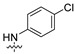	3.57 ± 0.89	6.96 ± 1.57	8.75 ± 1.59	6.58 ± 1.31	10.63 ± 2.46
**9**	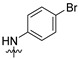	1.89 ± 0.59	5.15 ± 1.16	4.31 ± 0.96	4.75 ± 1.36	6.65 ± 1.57
**10**	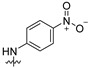	8.32 ± 2.06	12.15 ± 1.91	12.52 ± 2.83	15.65 ± 1.89	25.75 ± 2.20
**11**	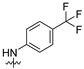	10.96 ± 1.93	15.68 ± 1.82	20.75 ± 2.16	25.37 ± 3.11	22.88 ± 2.69
**12**	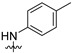	5.81 ± 2.41	10.63 ± 1.67	15.32 ± 1.53	8.56 ± 1.72	19.35 ± 2.38
**13**	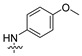	4.98 ± 1.26	8.36 ± 1.92	6.83 ± 1.29	5.69 ± 1.68	>30
**14**	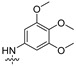	2.12 ± 0.97	3.21 ± 2.28	5.35 ± 1.82	8.72 ± 1.53	13.17 ± 1.86
**15**	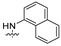	18.86 ± 2.82	>30	15.37 ± 1.92	12.15 ± 1.59	10.39 ± 1.88
**16**	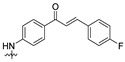	2.02 ± 0.38	3.96 ± 0.92	2.75 ± 0.66	1.98 ± 0.26	3.67 ± 0.93
**17**	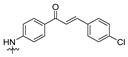	1.39 ± 0.26	2.37 ± 0.35	1.89 ± 0.67	1.46 ± 0.16	4.75 ± 0.81
**18**	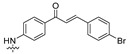	0.85 ± 0.14	1.61 ± 0.20	1.26 ± 0.18	1.54 ± 0.38	2.12 ± 0.52
**19**	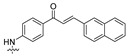	1.56 ± 0.92	1.96 ± 0.56	1.39 ± 0.25	2.55 ± 0.49	2.61 ± 0.59
5-Fu	---	4.11 ± 0.72	4.51 ± 1.23	3.68 ± 1.06	5.12 ± 1.73	2.68 ± 0.86

The data were generated from the MTT assay, and values were averaged from independent experiments.

**Table 2:** Inhibitory activity of compounds **6**–**19** toward GES-1.

Compound	IC_50_ (μM) GES-1	Compound	IC_50_ (μM) GES-1
**6**	>30	**14**	19.62 ± 2.58
**7**	20.82 ± 3.29	**15**	>30
**8**	>30	**16**	18.75 ± 1.56
**9**	18.57 ± 1.72	**17**	23.42 ± 2.83
**10**	>30	**18**	26.75 ± 1.85
**11**	25.41 ± 3.51	**19**	19.82 ± 2.21
**12**	24.28 ± 1.96	**5-Fu**	3.26 ± 1.07
**13**	21.31 ± 2.79		

The data were generated from the MTT assay, and values were averaged from independent experiments.

## Data Availability

Data are contained within the article.

## Supplementary Materials

The following supporting information can be downloaded at: https://www.mdpi.com/article/10.3390/molecules27123906/s1, Figures S1–S42: ^1^H- and ^13^C-NMR and HRMS spectra of compounds **6**–**19**.

## Sample Availability

Sample of compound **18** is available from the authors.
