# Supplementary material for: Discovery of Novel Quinazoline Derivatives as Potent Antitumor Agents
Source: Molecules. 2022 Jun 17;27(12):3906. doi: 10.3390/molecules27123906 (PMC9230651; doi:10.3390/molecules27123906)
Supplement: Supplementary file 1 [file molecules-27-03906-s001.zip › molecules-1730525-supplementary.pdf]

Supplementary Materials

Discovery of Novel Quinazoline Derivatives as Potent Antitumor Agents

Zhenxi Niu [1], Shuli Ma [1], Lei Zhang [2], Qibing Liu [2,3,*] and Shengnan Zhang [1,*]

1 Department of Pharmacy, Children's Hospital Affiliated to Zhengzhou University, Henan Children's
Hospital, Zhengzhou Children's Hospital, Zhengzhou 450018, China; zzuzhenxiniu@outlook.com (Z.N.); 13633860971@163.com (S.M.)

2 Department of Pharmacy, The First Affiliated Hospital of Hainan Medical University, Haikou 570100, China; zl0898@hainmc.edu.cn

3 Martinos Center for Biomedical Imaging, Massachusetts General Hospital and Harvard Medical School,
149 Thirteenth Street, Suite 2301, Boston, MA 02129, USA

* Correspondence: qibing.liu@hainmc.edu.cn (Q.L.); matensen@gs.zzu.edu.cn (S.Z.)

1. ^{1}H-NMR, ^{13}C-NMR, and HRMS spectra of 6-19

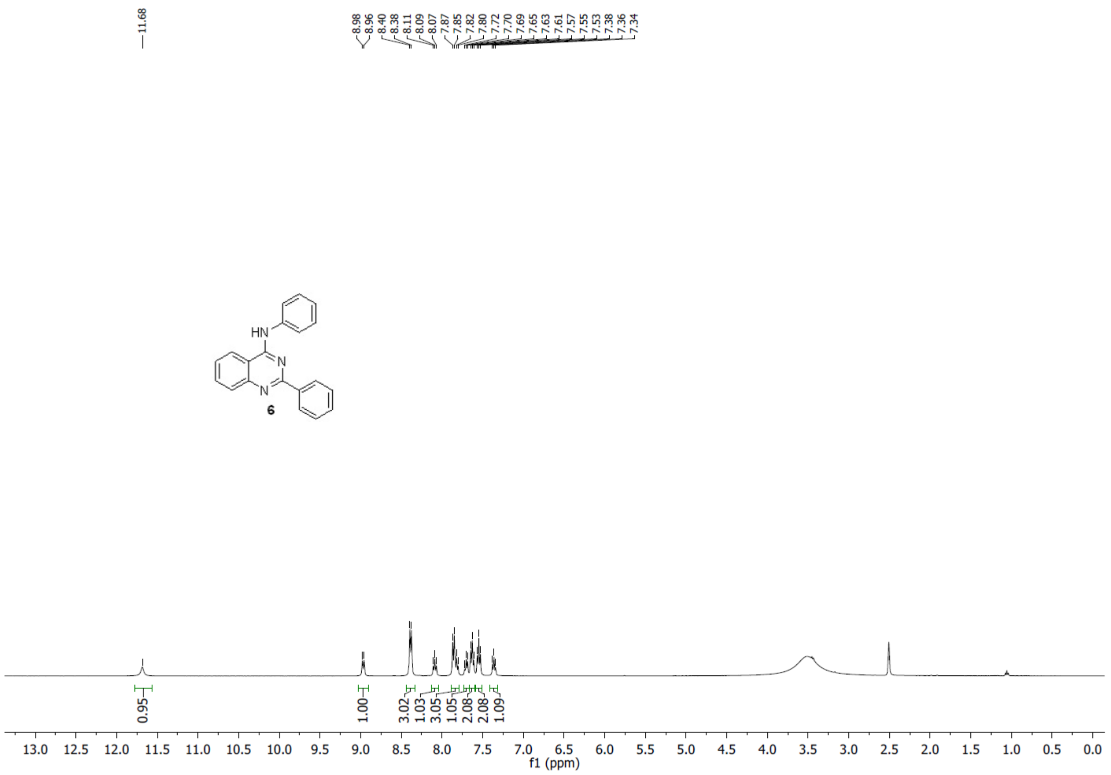

Figure S1 ^{1}H NMR spectra of 6

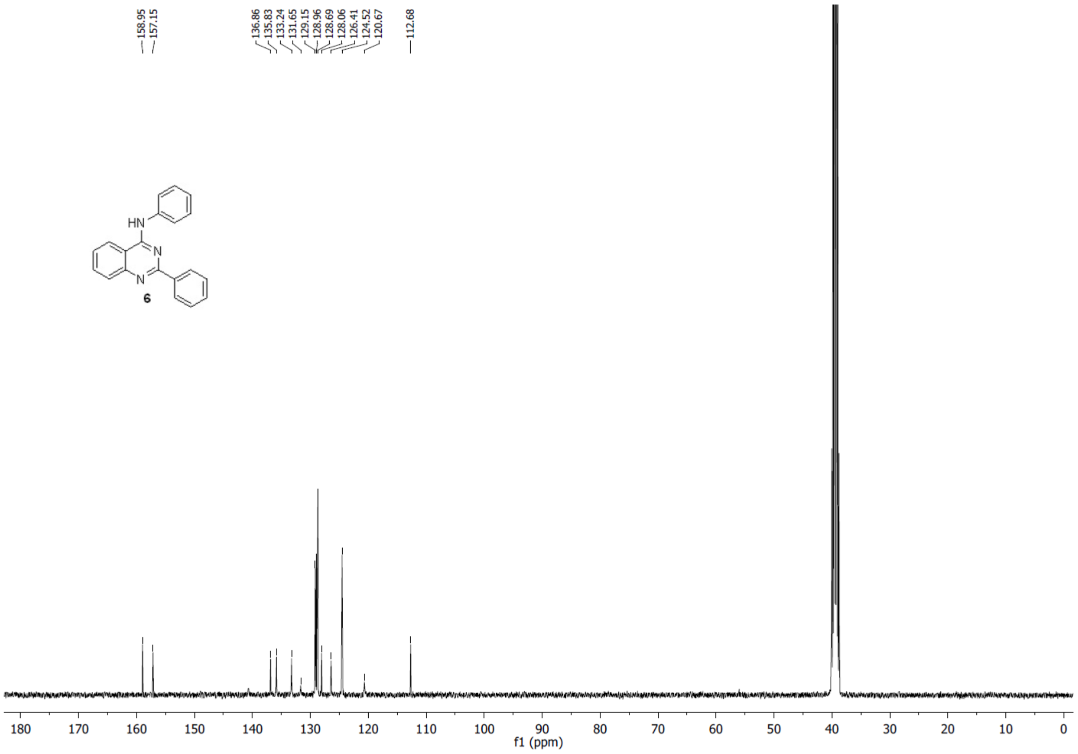

Figure S2 ^{13}C NMR spectra of 6

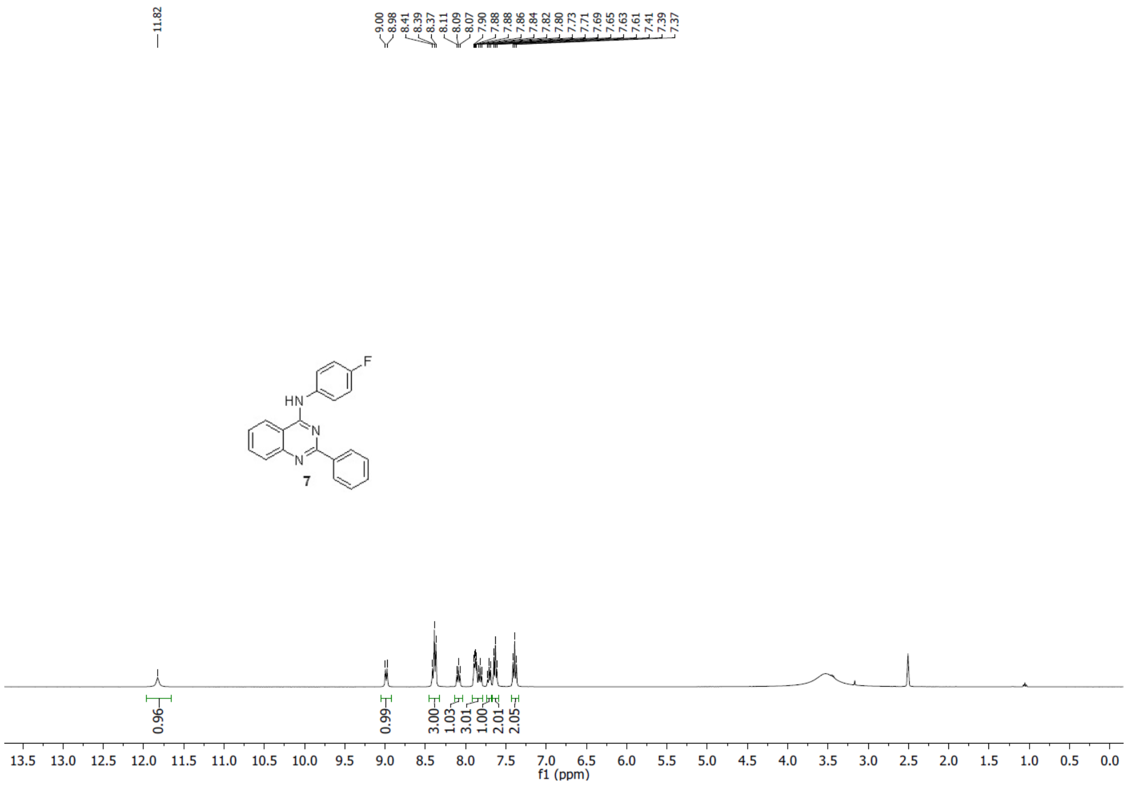

Figure S3 ^{1}H NMR spectra of 7

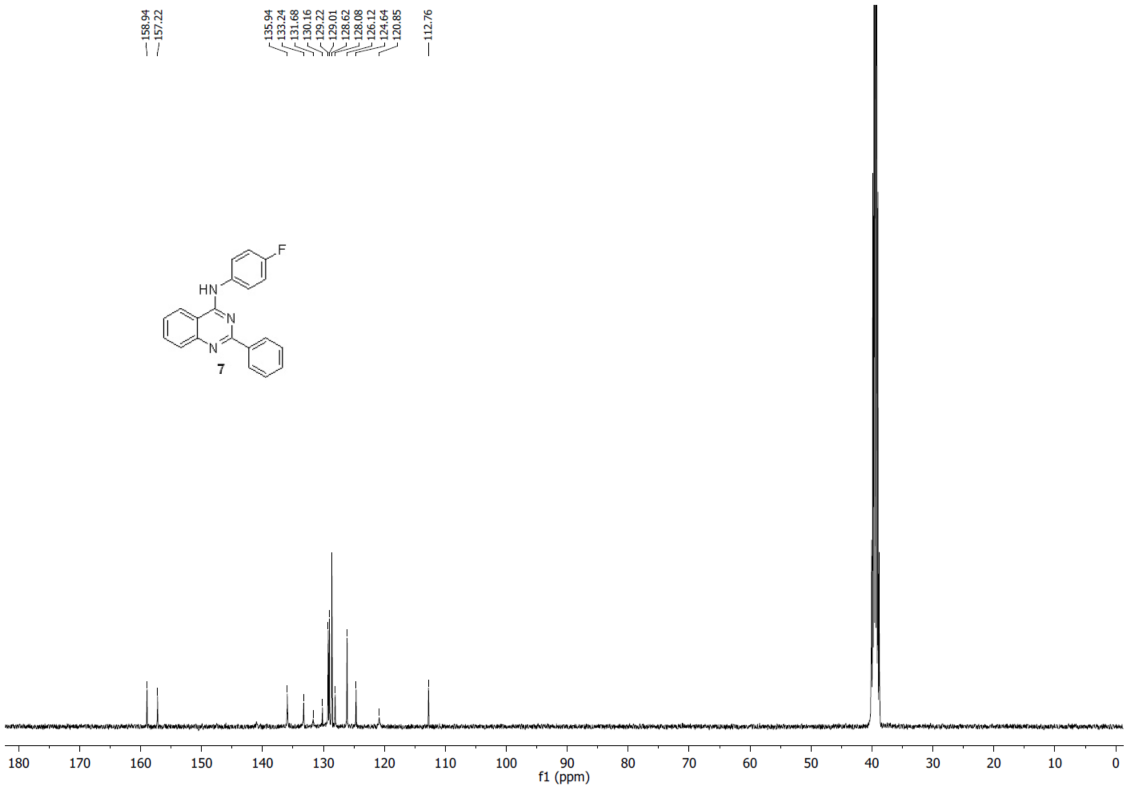

Figure S4 ^{13}C NMR spectra of 7

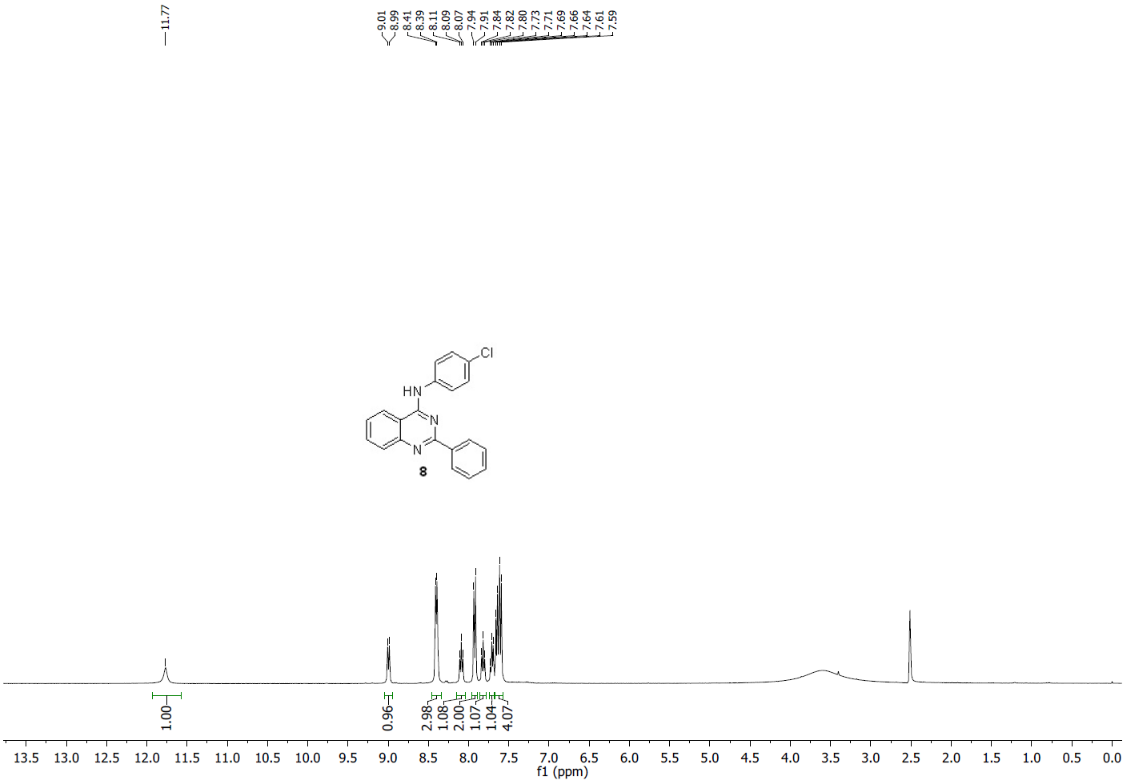

Figure S5 ^{1}H NMR spectra of 8

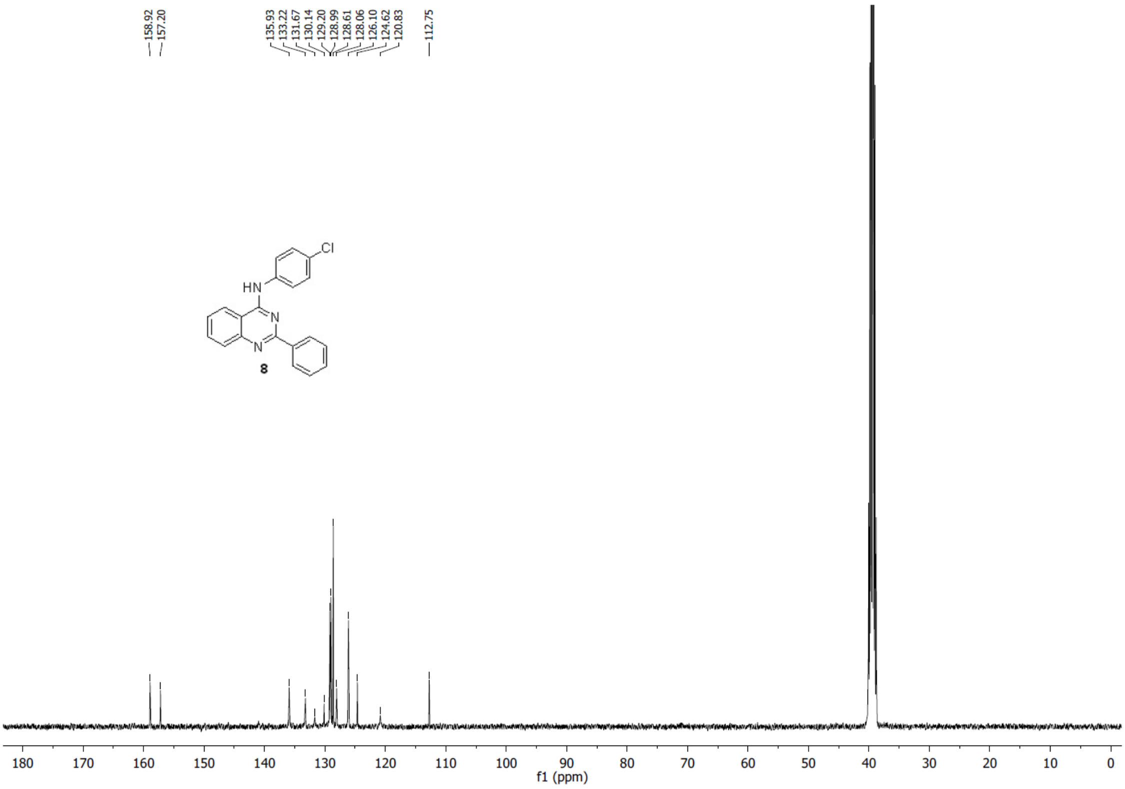

Figure S6 ^{13}C NMR spectra of 8

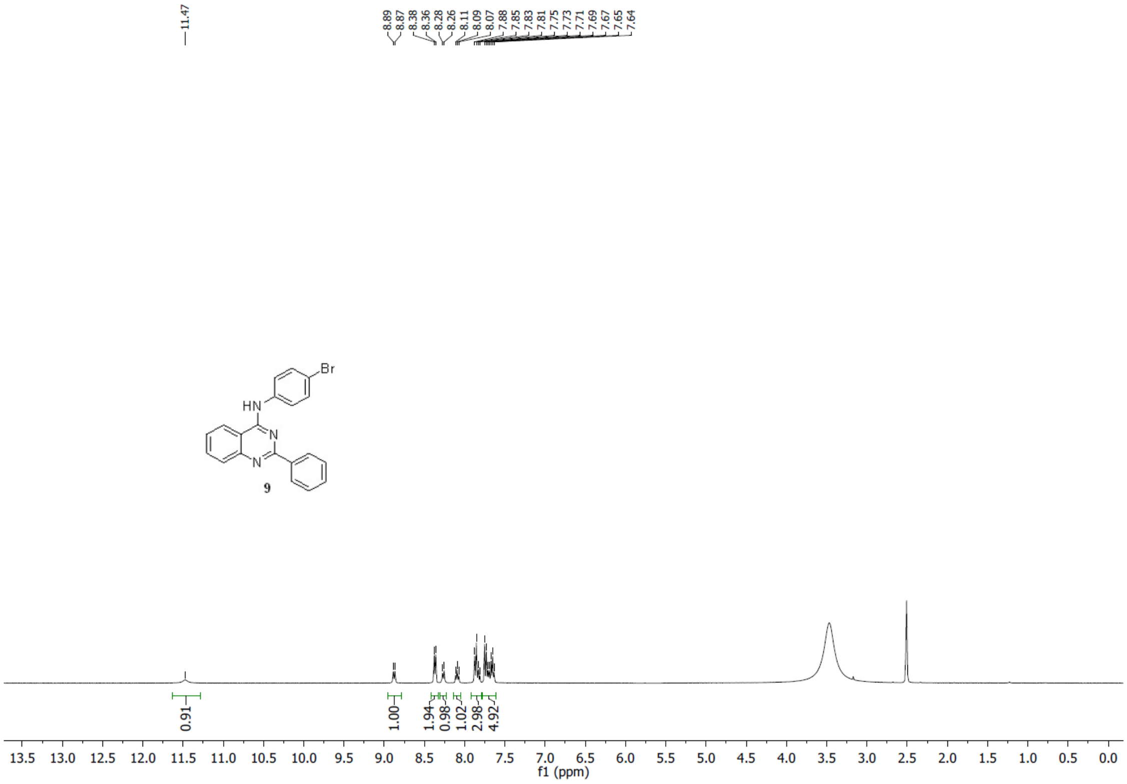

Figure S7 ^{1}H NMR spectra of 9

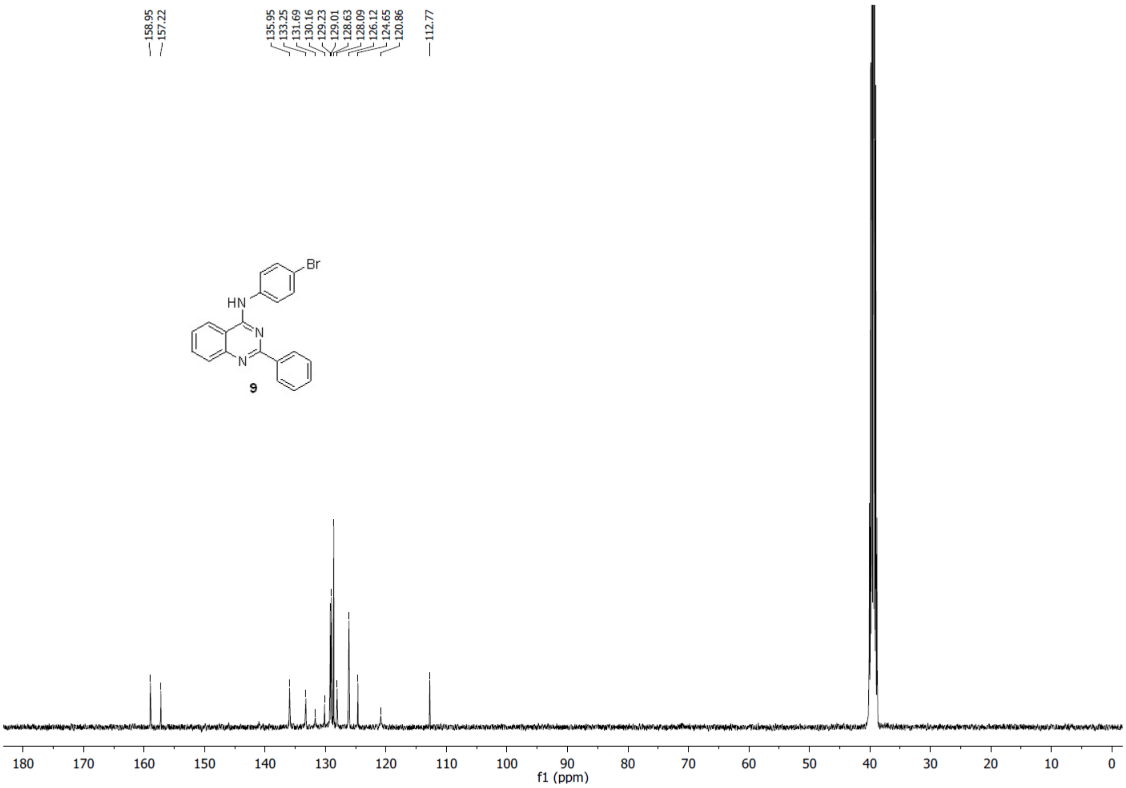

Figure S8 ^{13}C NMR spectra of 9

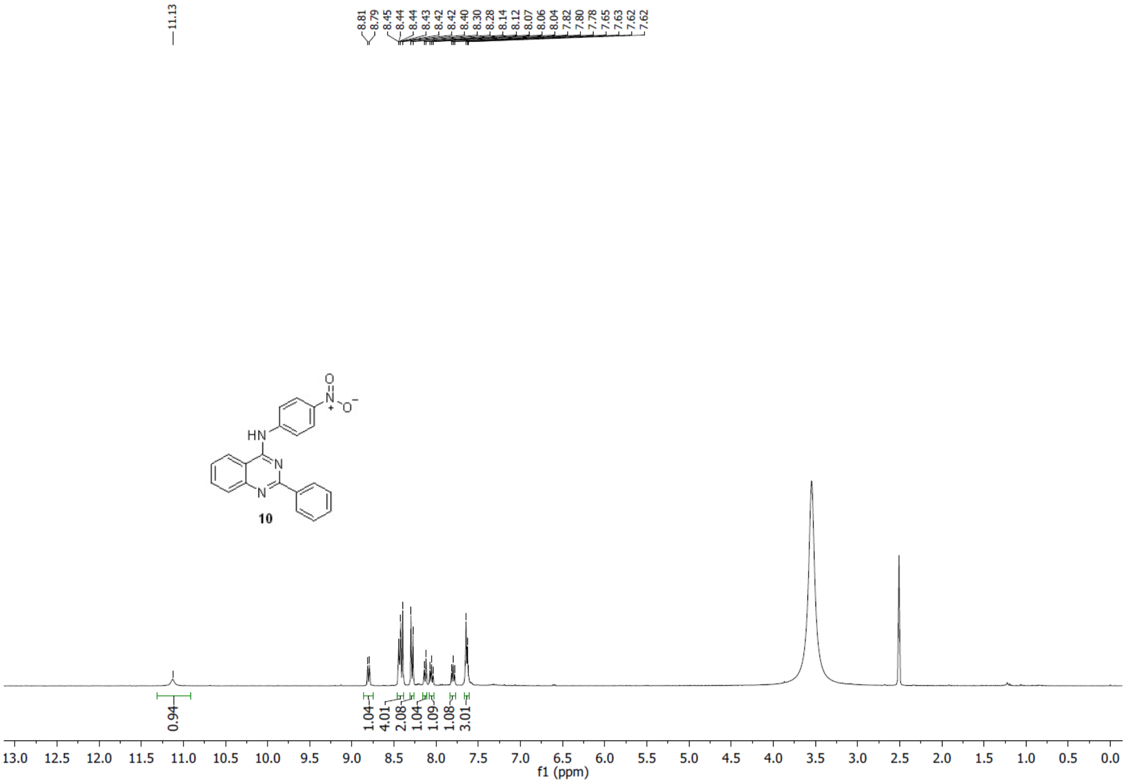

Figure S9 ^{1}H NMR spectra of 10

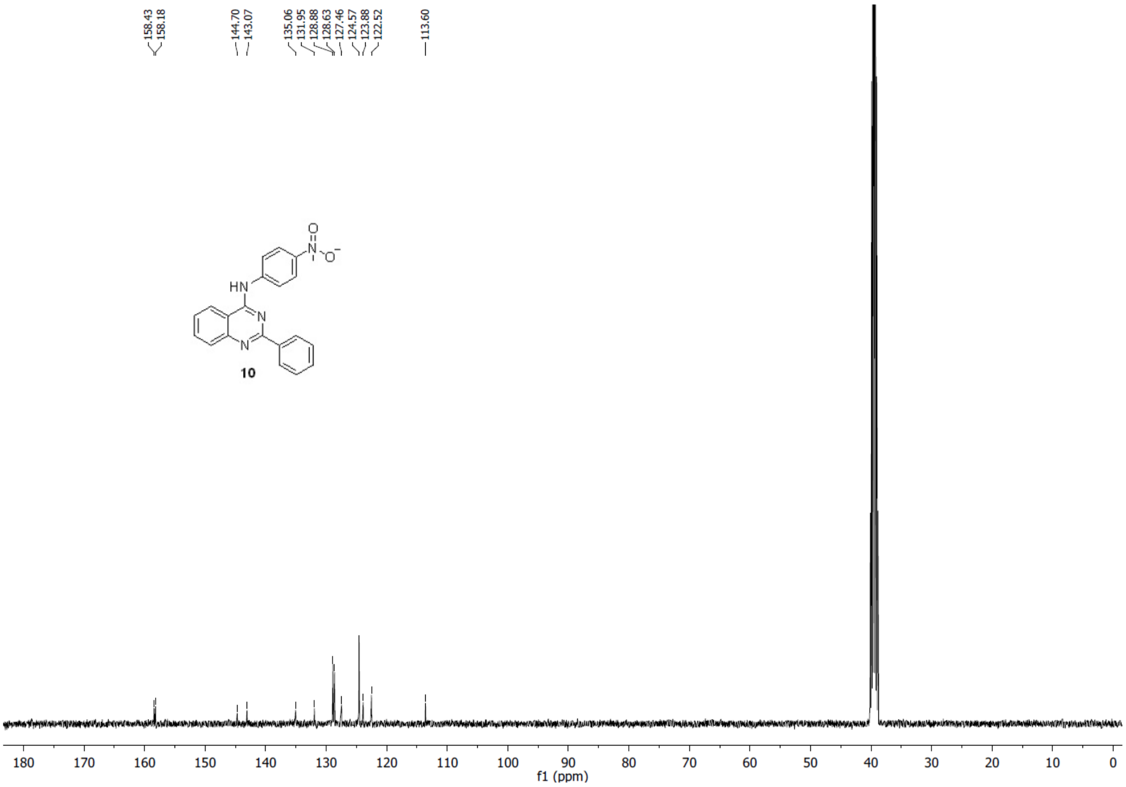

Figure S10 ^{13}C NMR spectra of 10

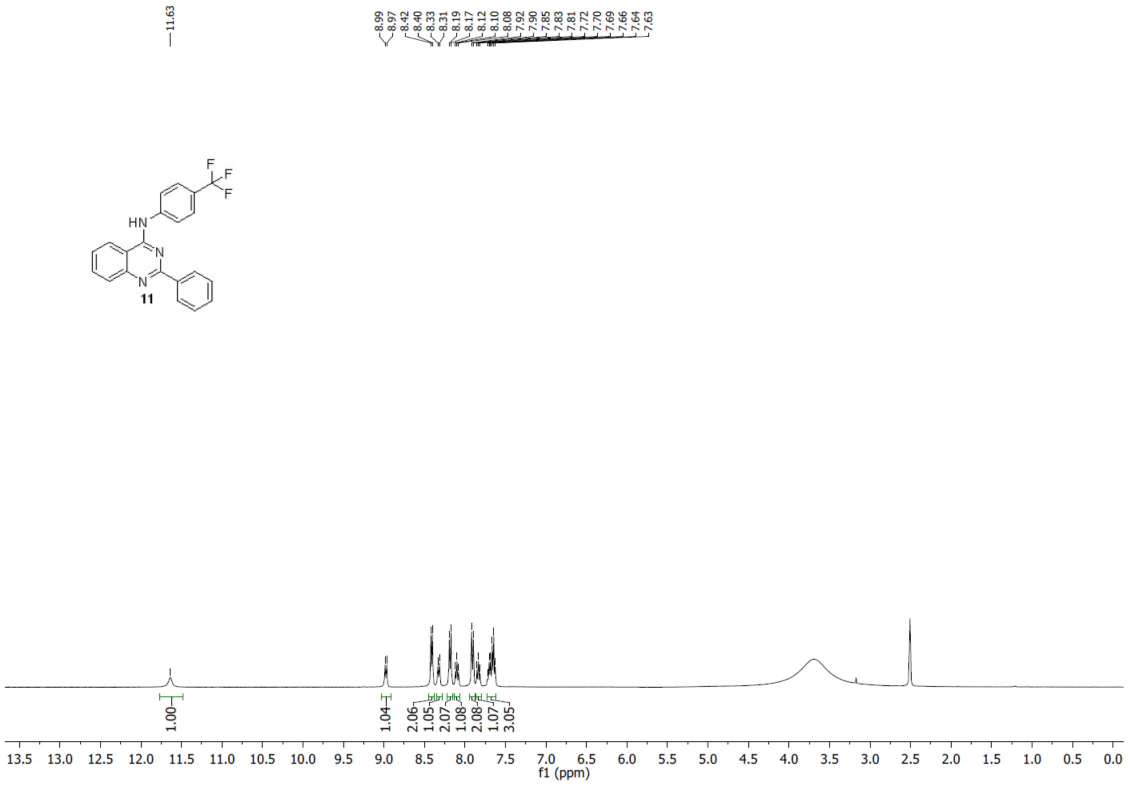

Figure S11 ^{1}H NMR spectra of 11

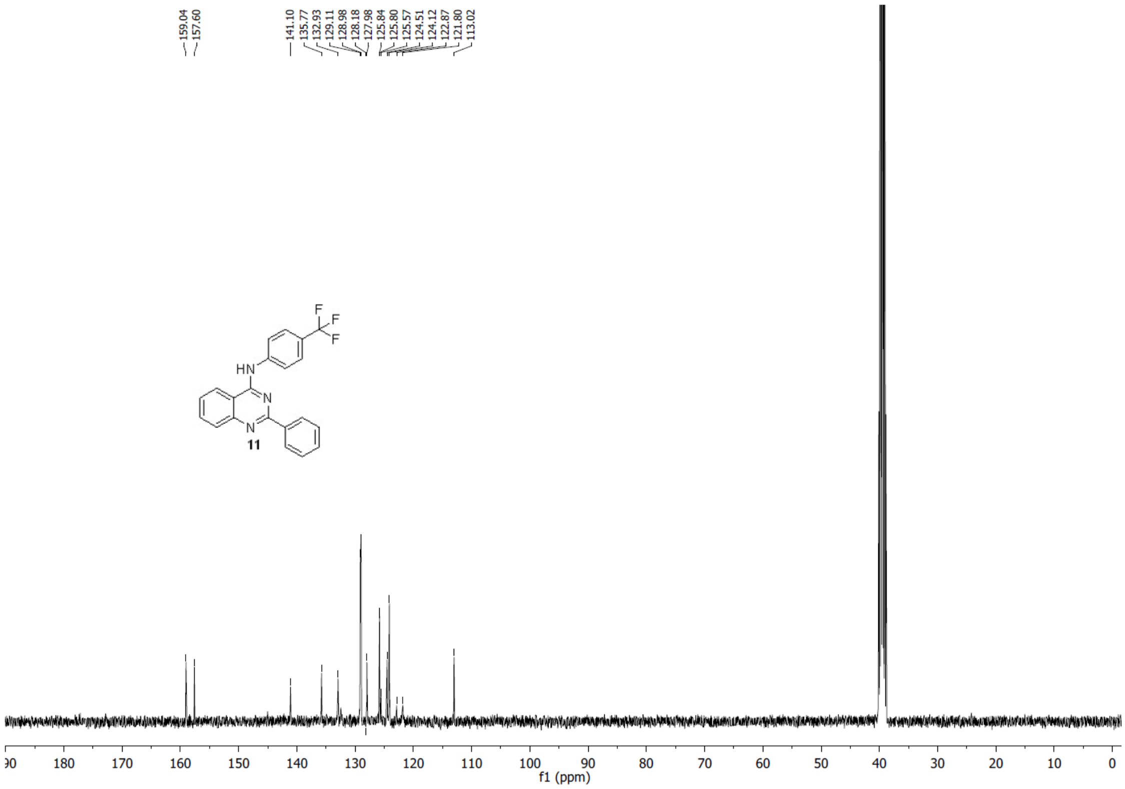

Figure S12 ^{13}C NMR spectra of 11

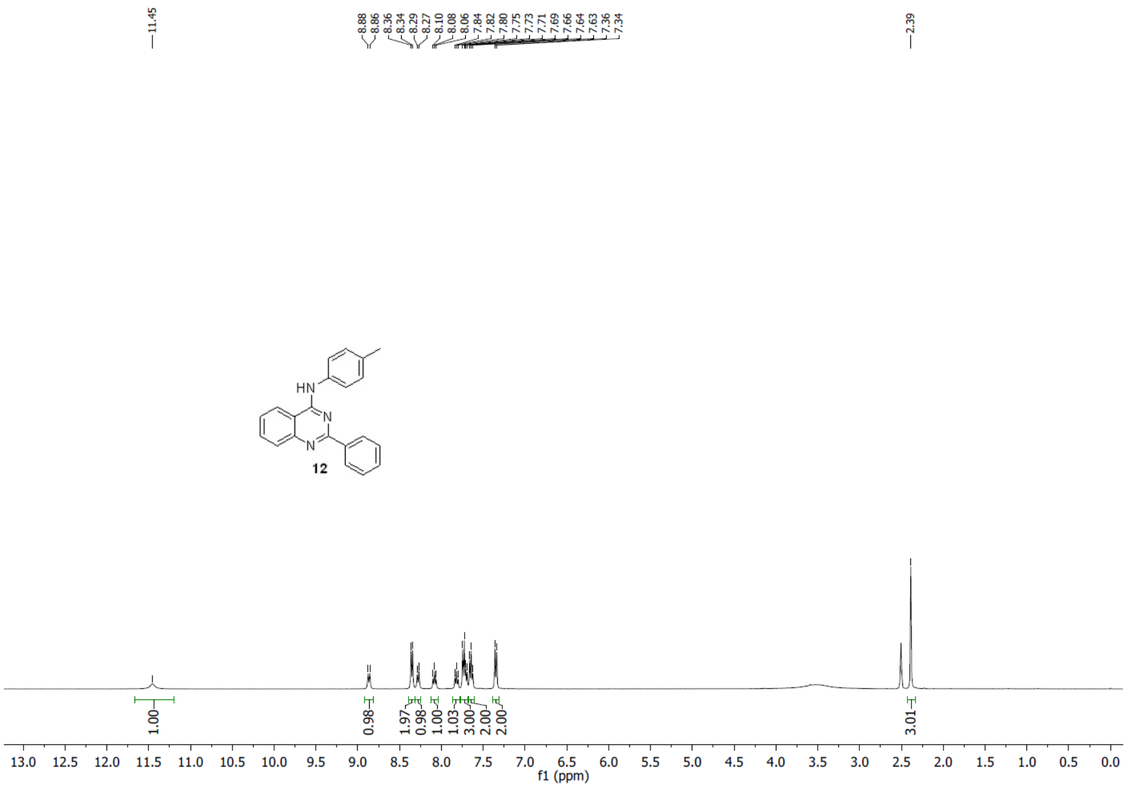

Figure S13 ^{1}H NMR spectra of 12

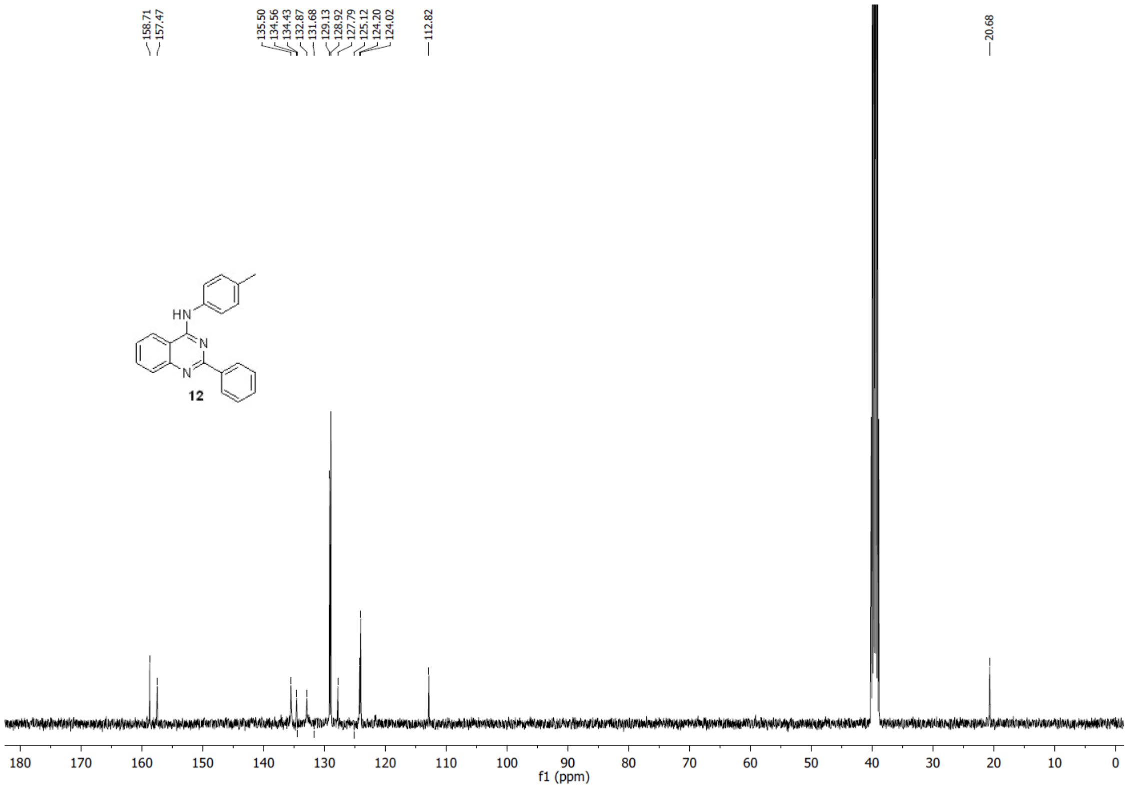

Figure S14 ^{13}C NMR spectra of 12

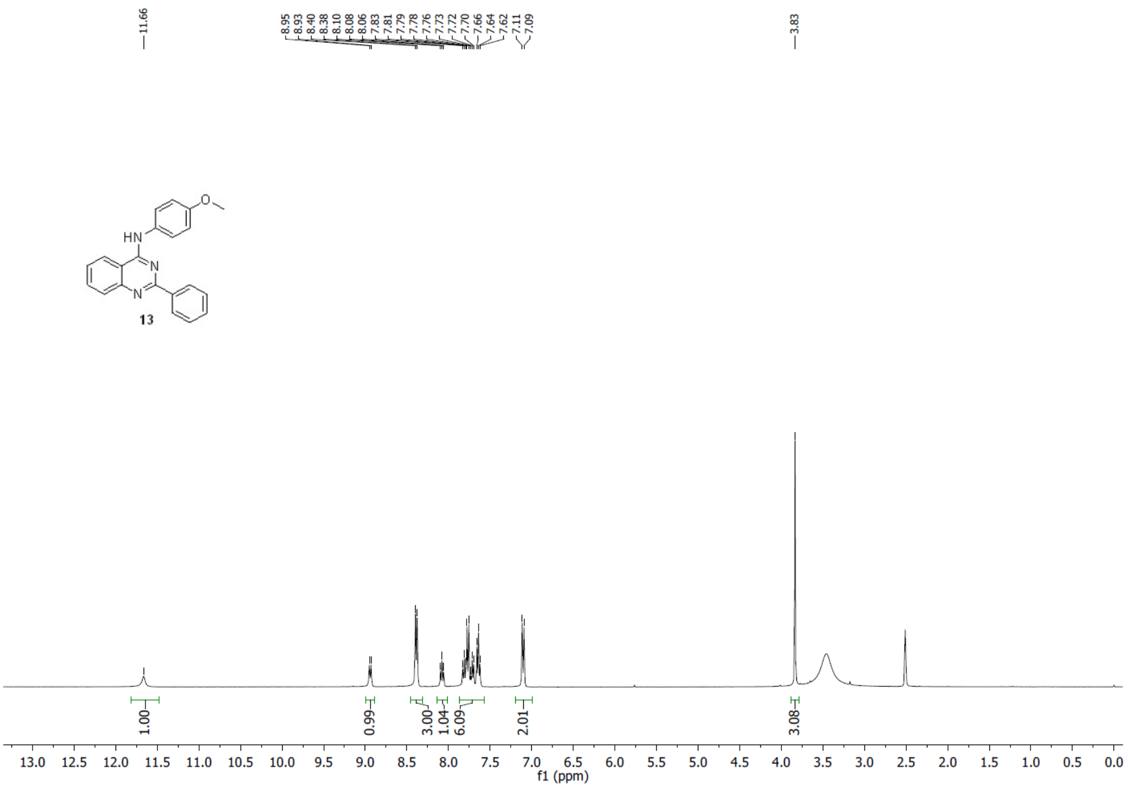

Figure S15 ^{1}H NMR spectra of 13

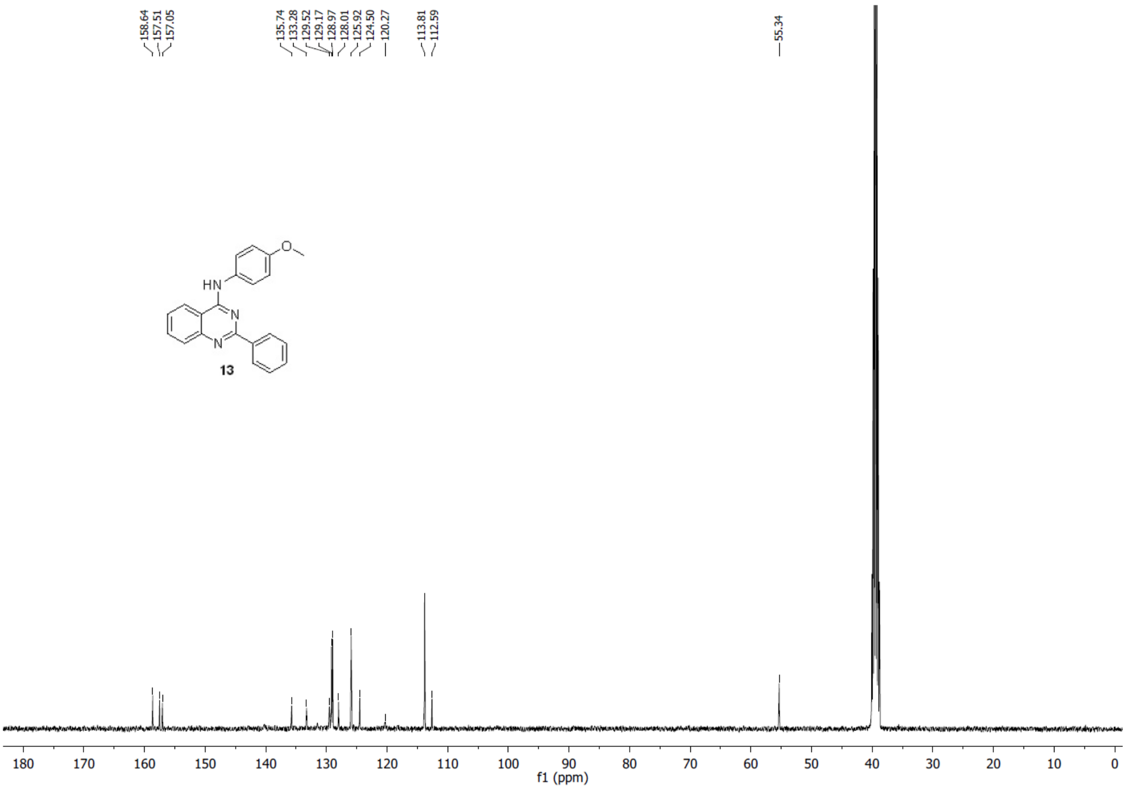

Figure S16 ^{13}C NMR spectra of 13

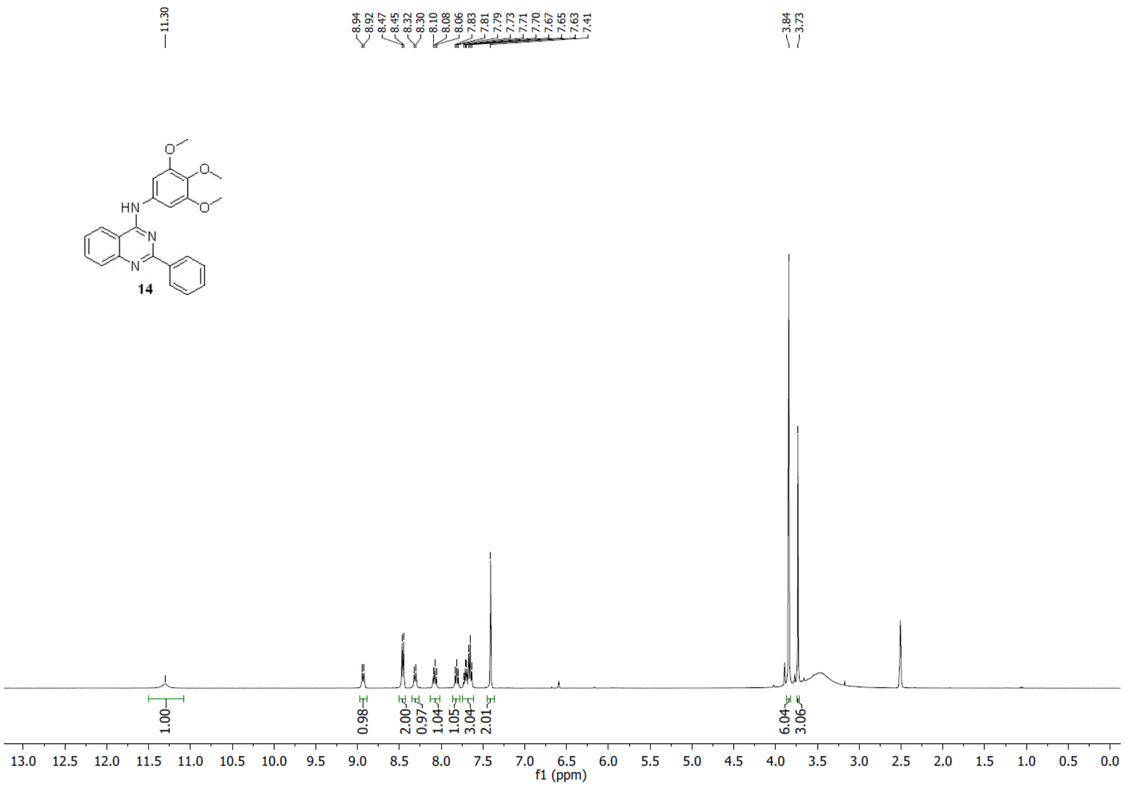

Figure S17 ^{1}H NMR spectra of 14

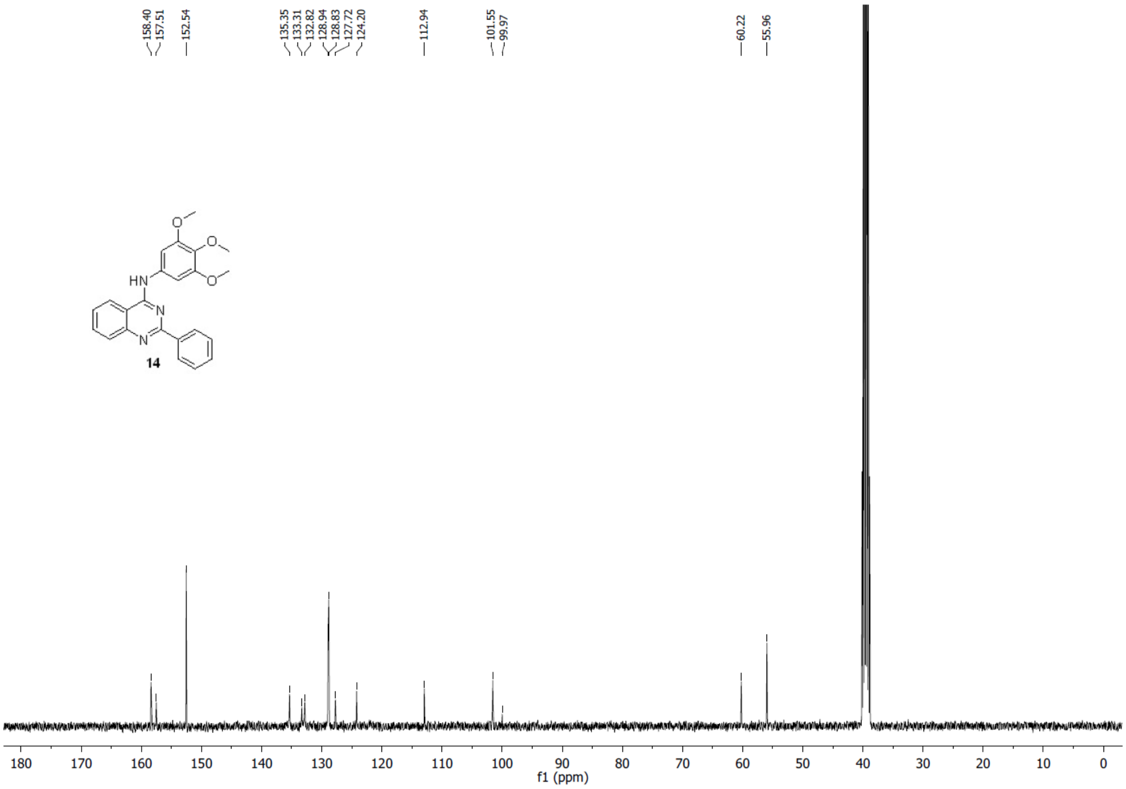

Figure S18 ^{13}C NMR spectra of 14

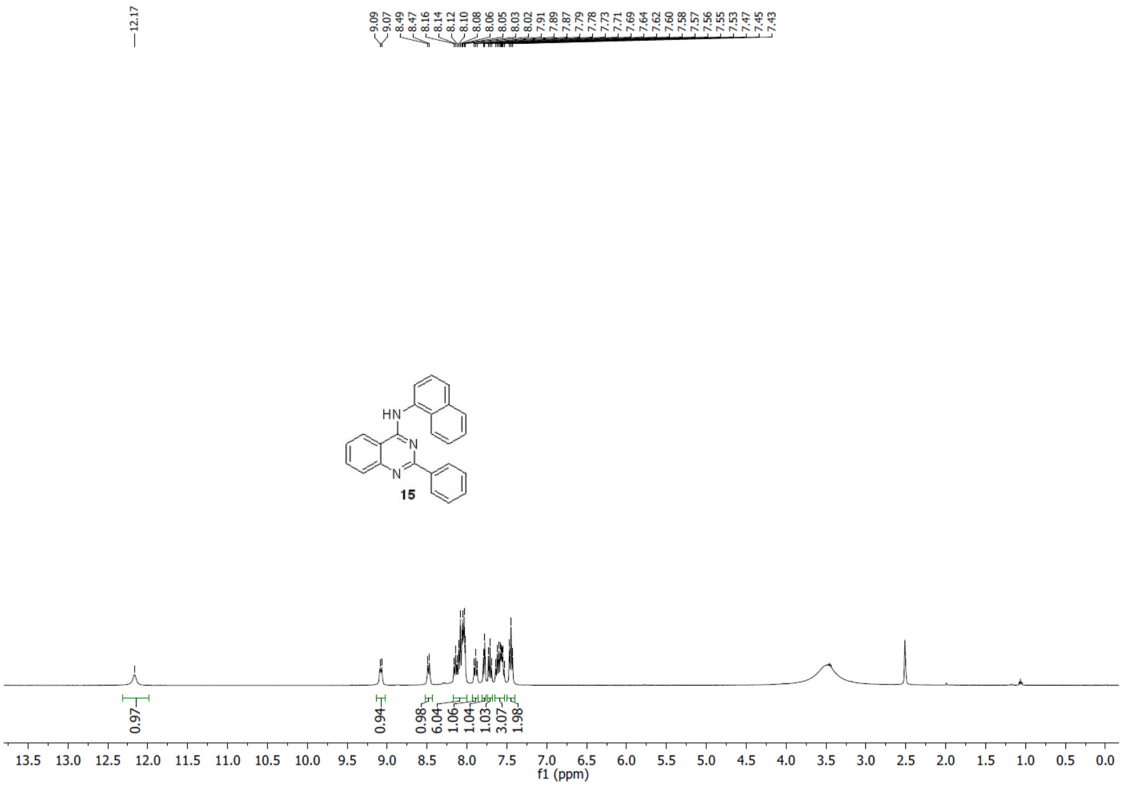

Figure S19 ^{1}H NMR spectra of 15

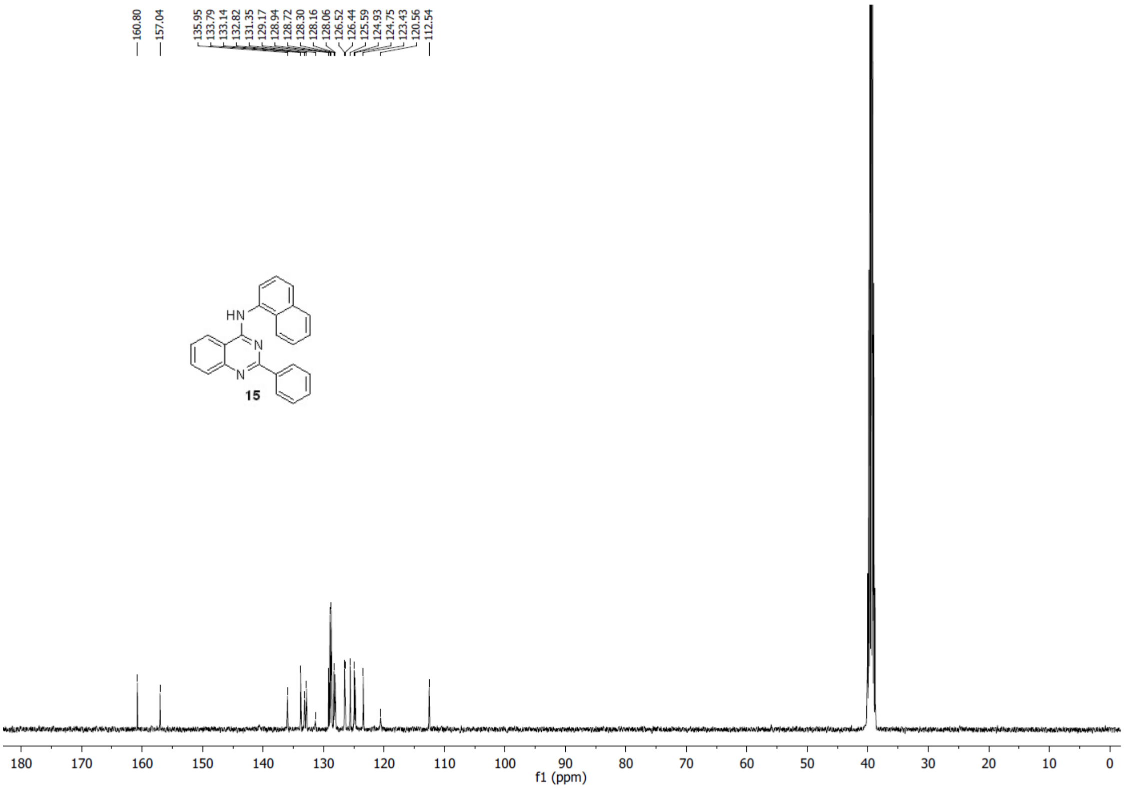

Figure S20 ^{13}C NMR spectra of 15

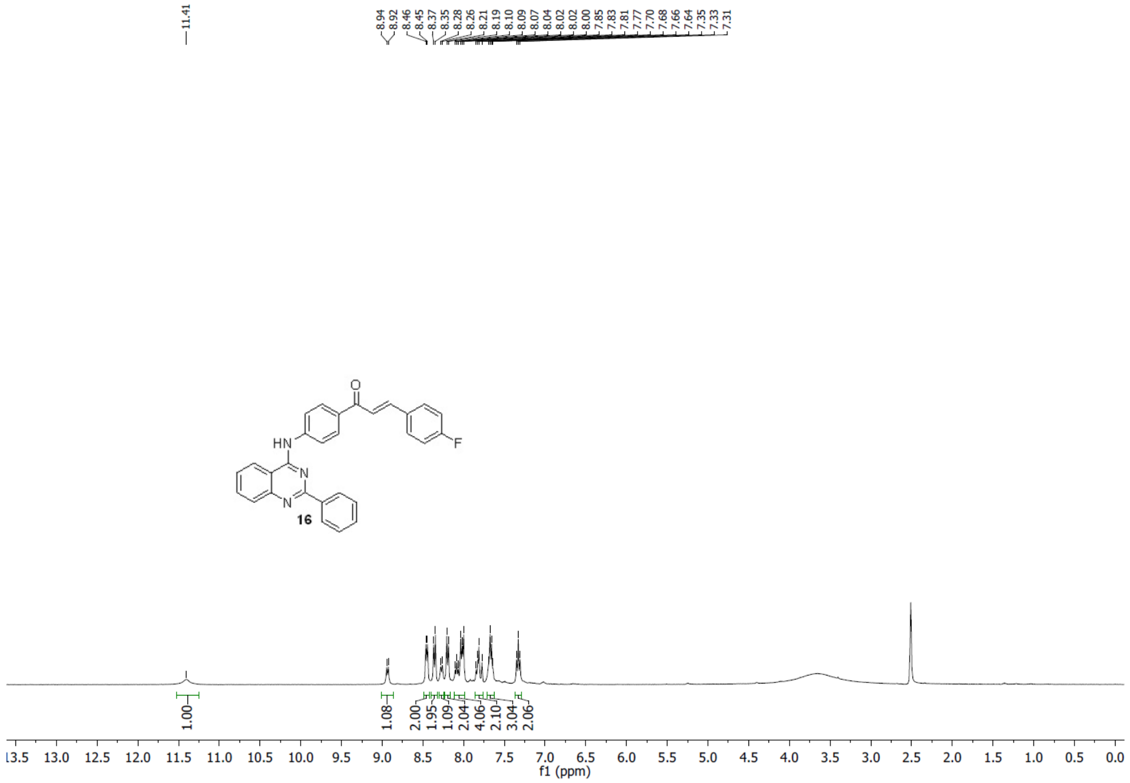

Figure S21 ^{1}H NMR spectra of 16

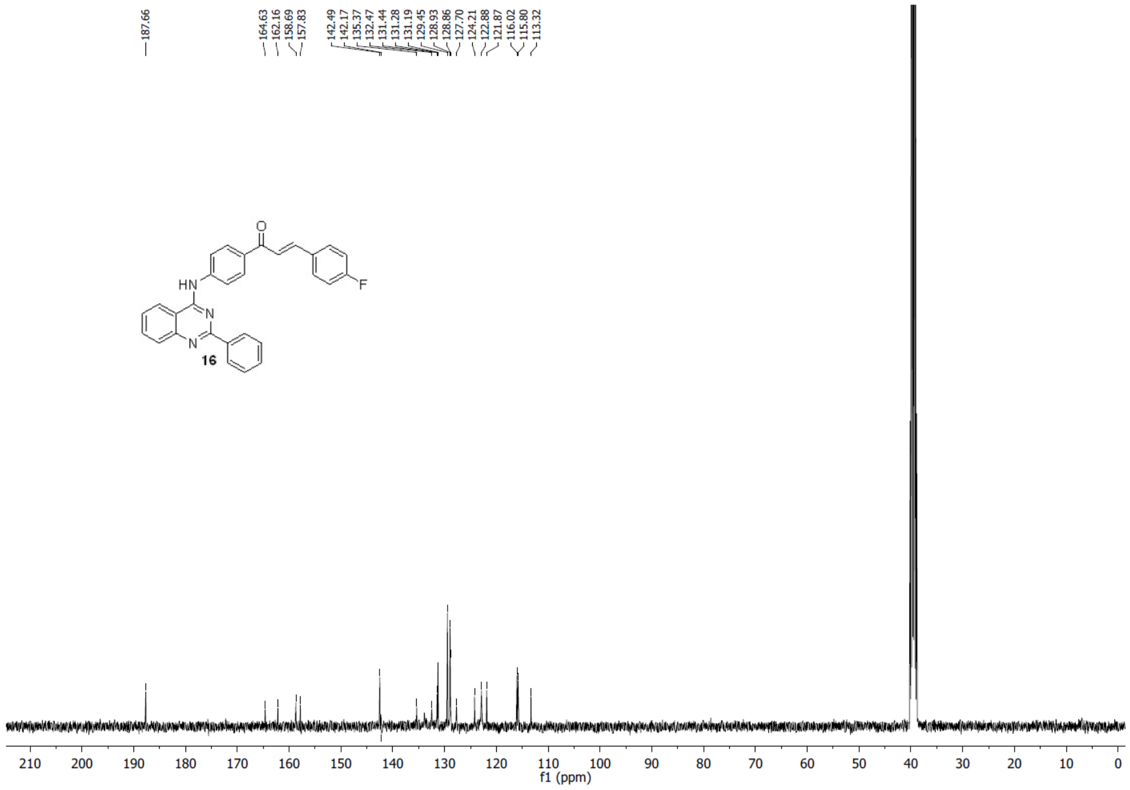

Figure S22 ^{13}C NMR spectra of 16

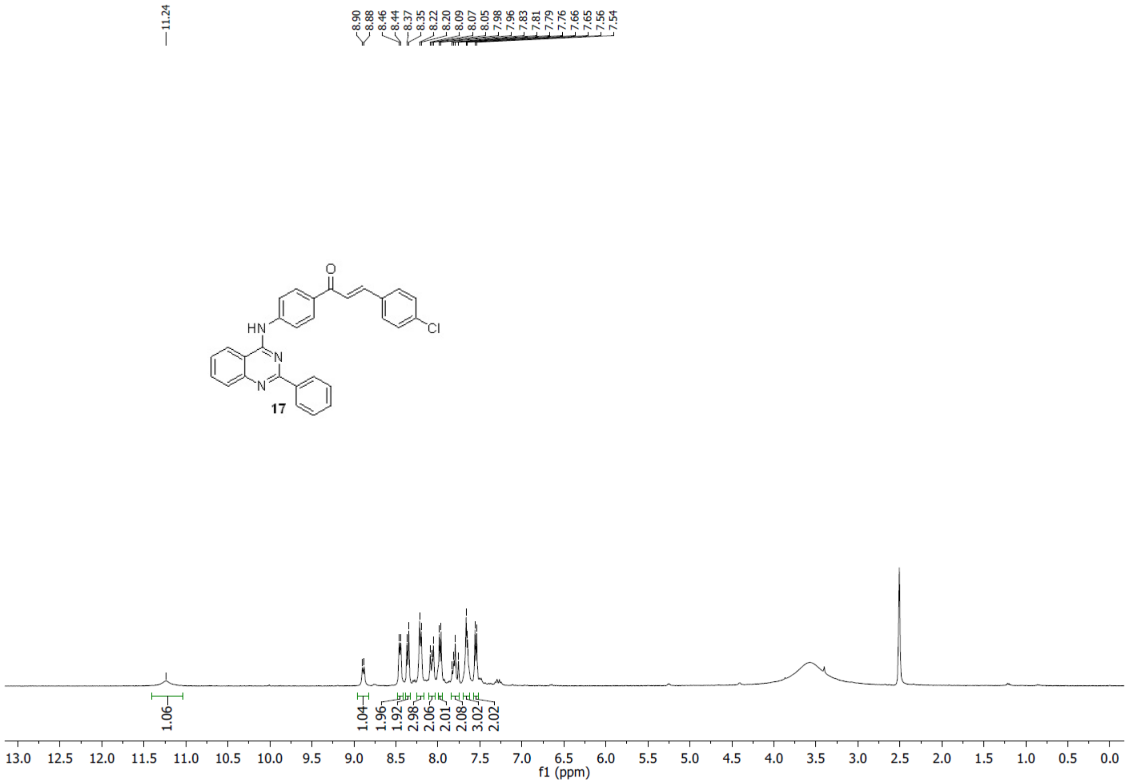

Figure S23 ^{1}H NMR spectra of 17

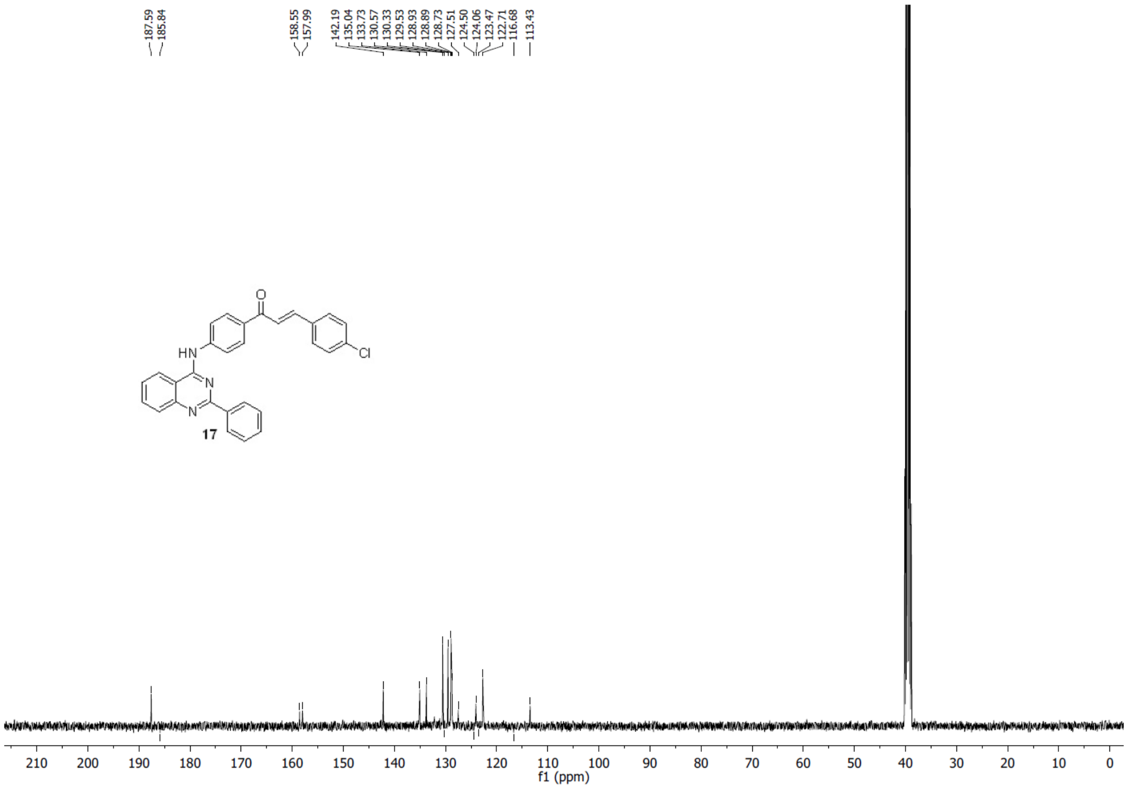

Figure S24 ^{13}C NMR spectra of 17

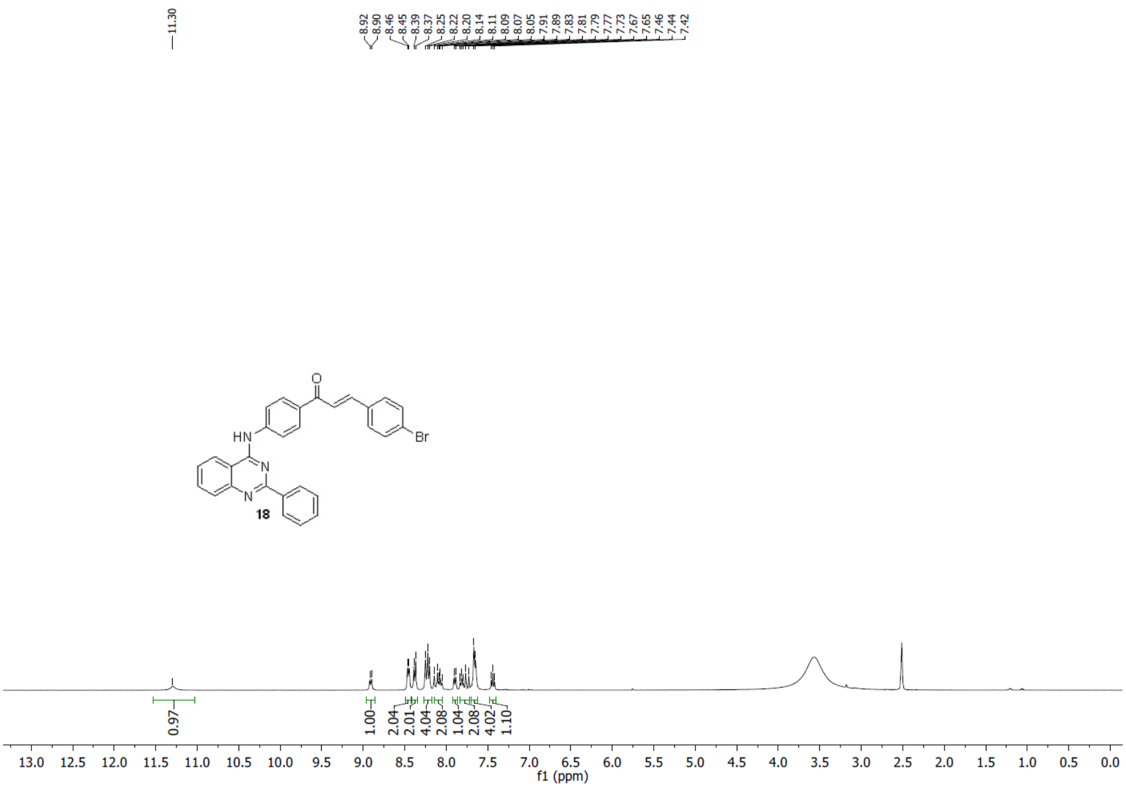

Figure S25 ^{1}H NMR spectra of 18

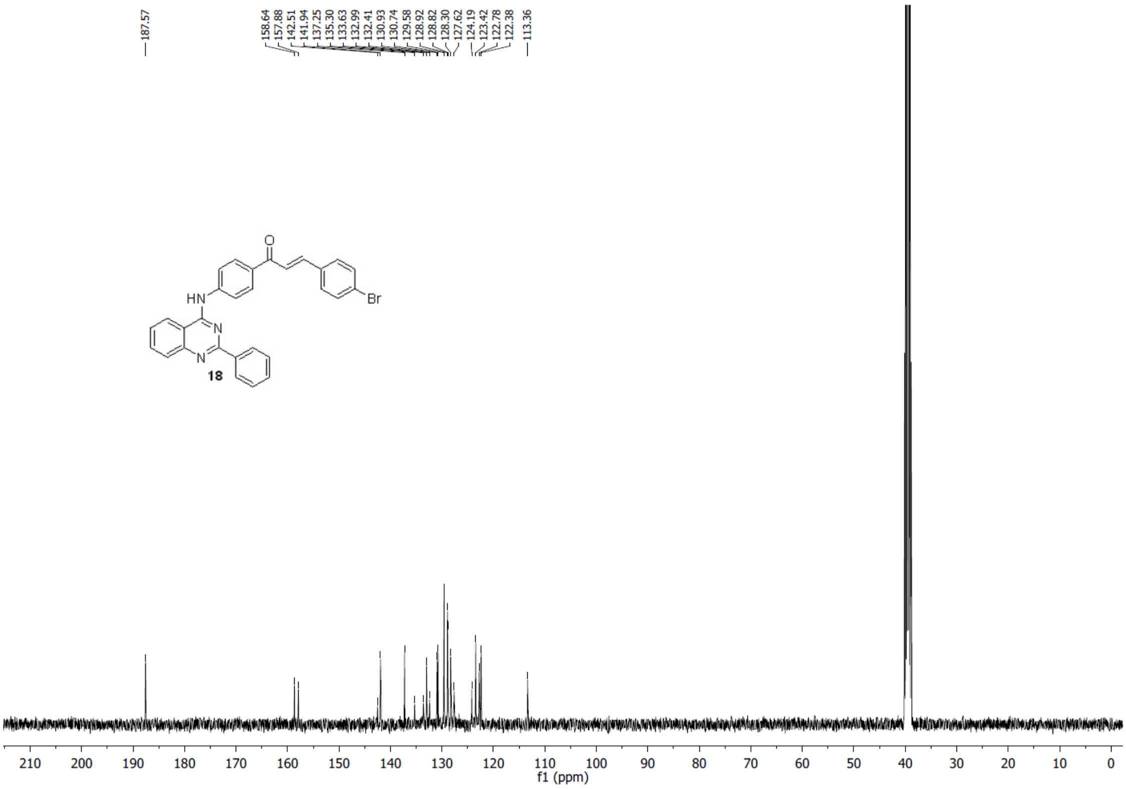

Figure S26 ^{13}C NMR spectra of 18

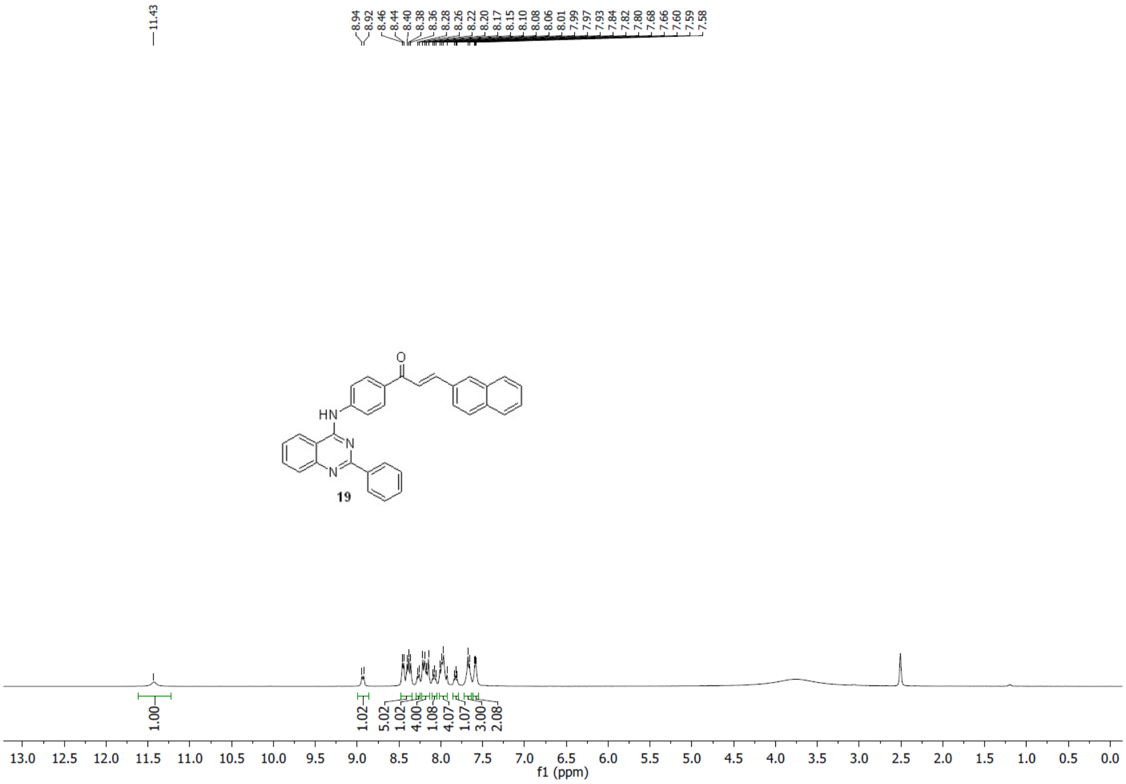

Figure S27 ^{1}H NMR spectra of 19

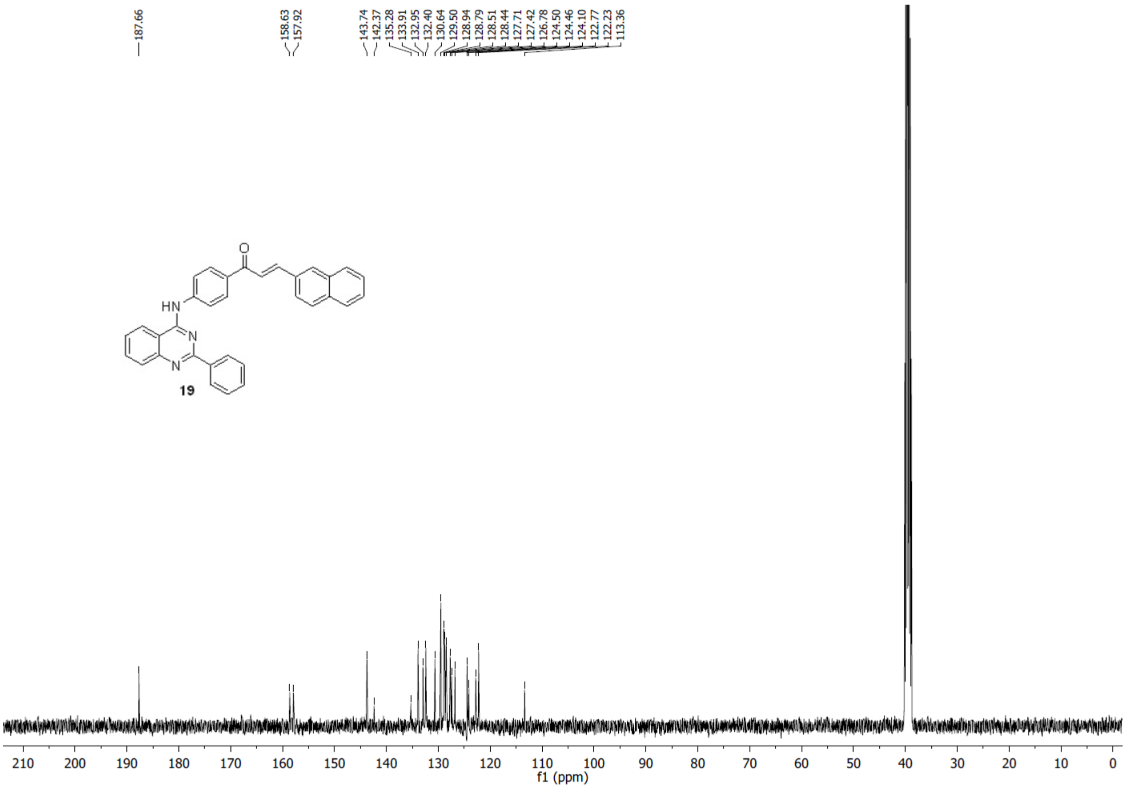

Figure S28 ^{13}C NMR spectra of 19

HRMS (ESI): m/z calcd for $C_{20}H_{16}N_3$ $(M+H)^+$, 298.1344; found, 298.1334.

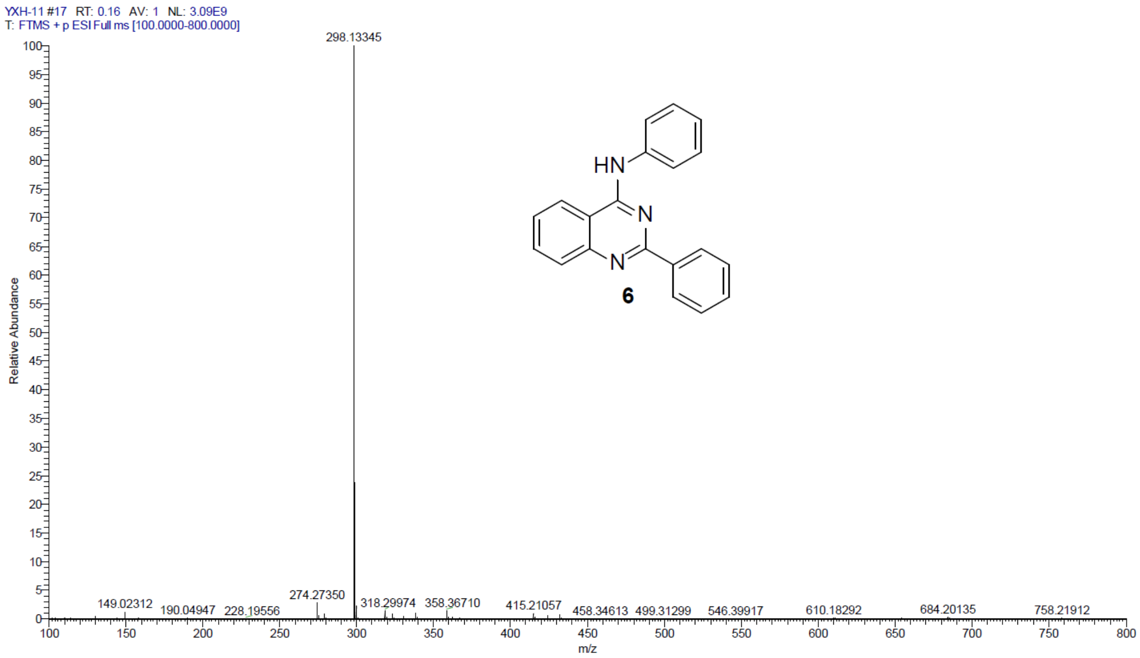

Figure S29 HRMS of 6

HRMS (ESI): m/z calcd for $C_{20}H_{15}FN_3$ $(M+H)^+$, 316.1250; found, 316.1240.

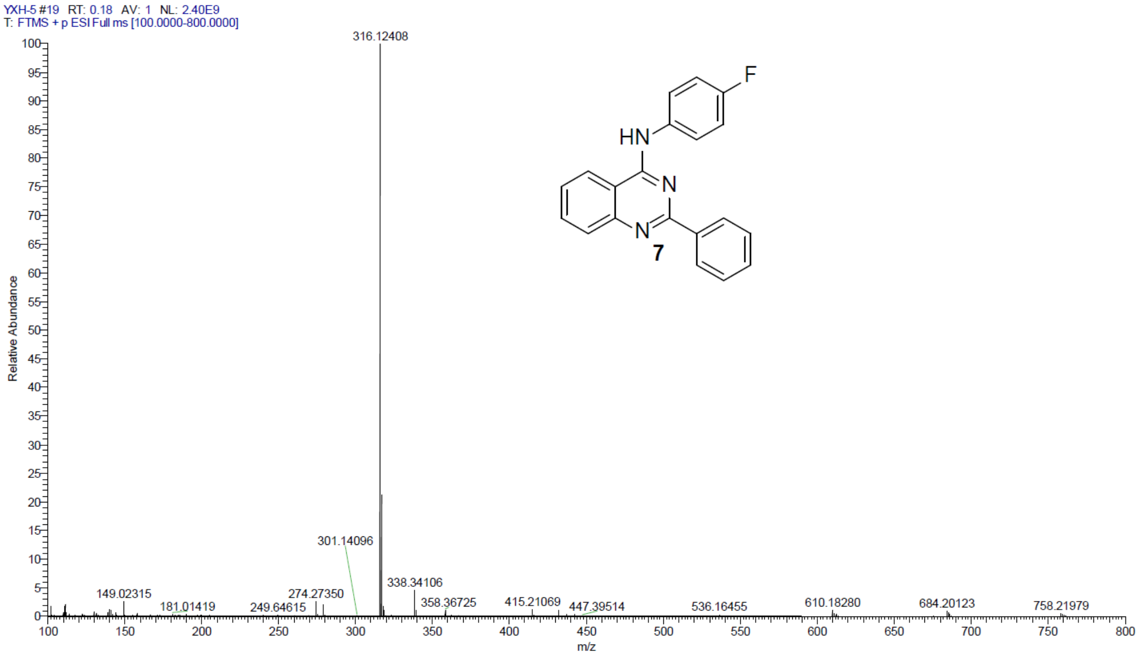

Figure S30 HRMS of 7

HRMS (ESI): m/z calcd for $C_{20}H_{15}ClN_3$ $(M+H)^+$, 332.0955; found, 332.0946.

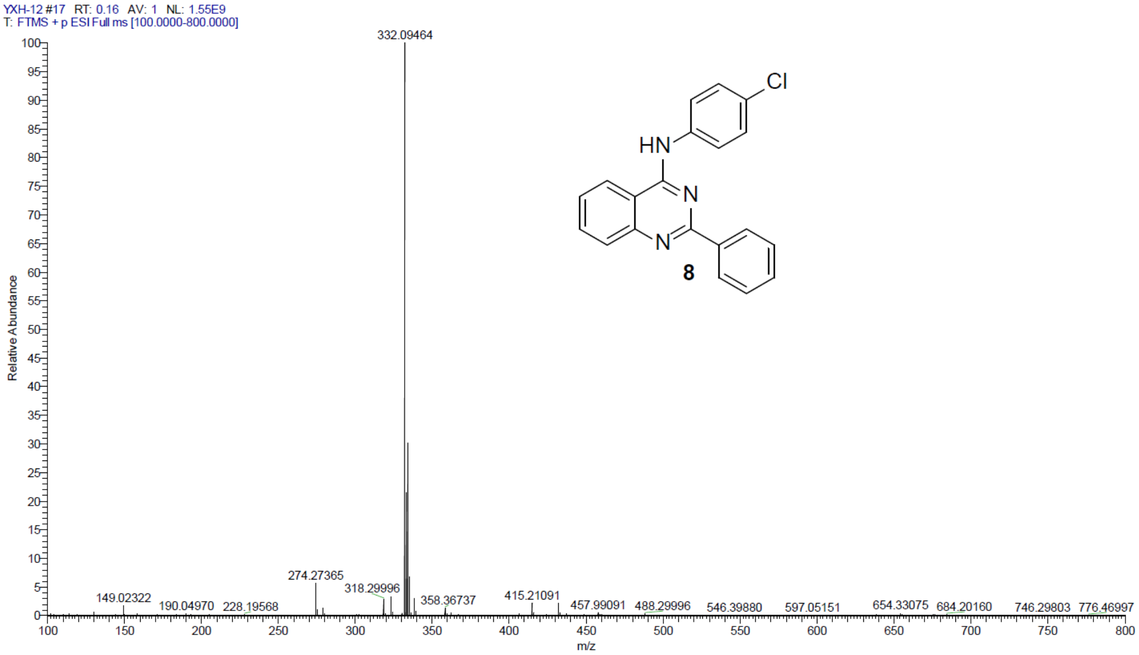

Figure S31 HRMS of 8

HRMS (ESI): m/z calcd for $C_{20}H_{15}BrN_3$ $(M+H)^+$, 376.0449; found, 376.0442.

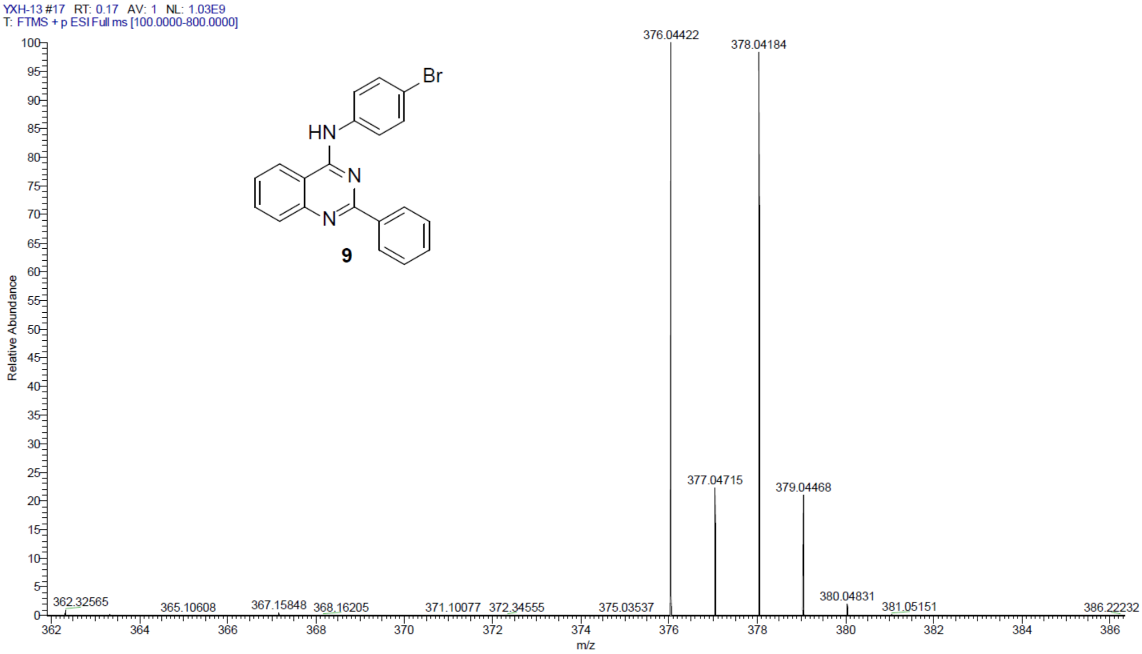

Figure S32 HRMS of 9

HRMS (ESI): m/z calcd for $C_{20}H_{15}N_4O_2$ $(M+H)^+$, 343.1195; found, 343.1186.

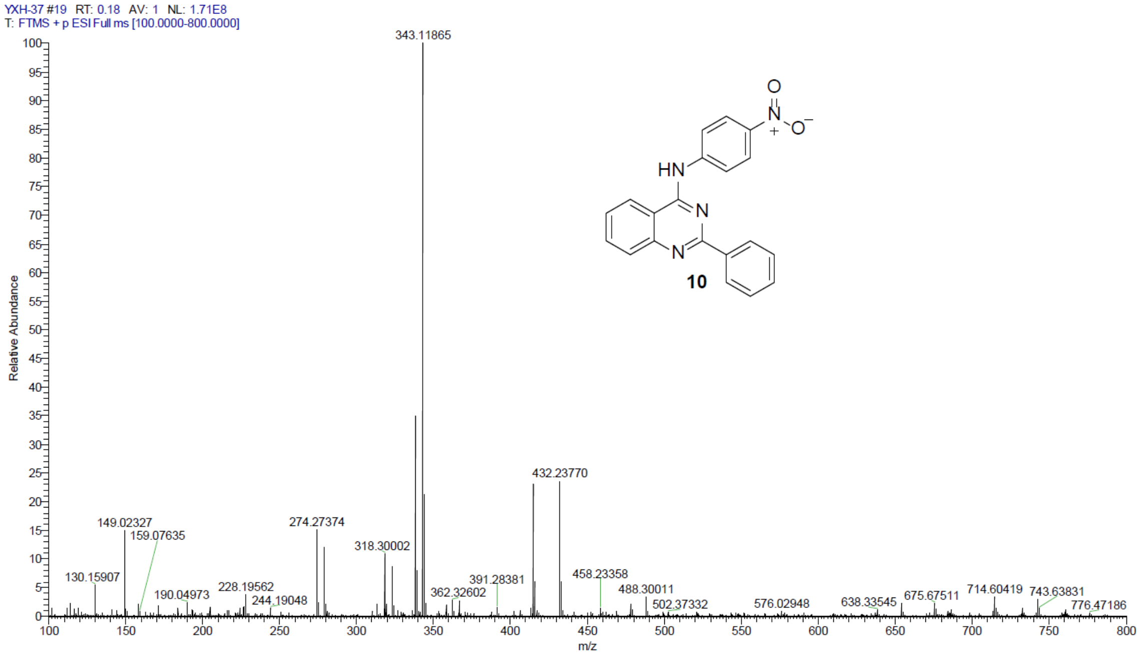

Figure S33 HRMS of 10

HRMS (ESI): m/z calcd for $C_{21}H_{15}F_3N_3$ $(M+H)^+$, 366.1218; found, 366.1208.

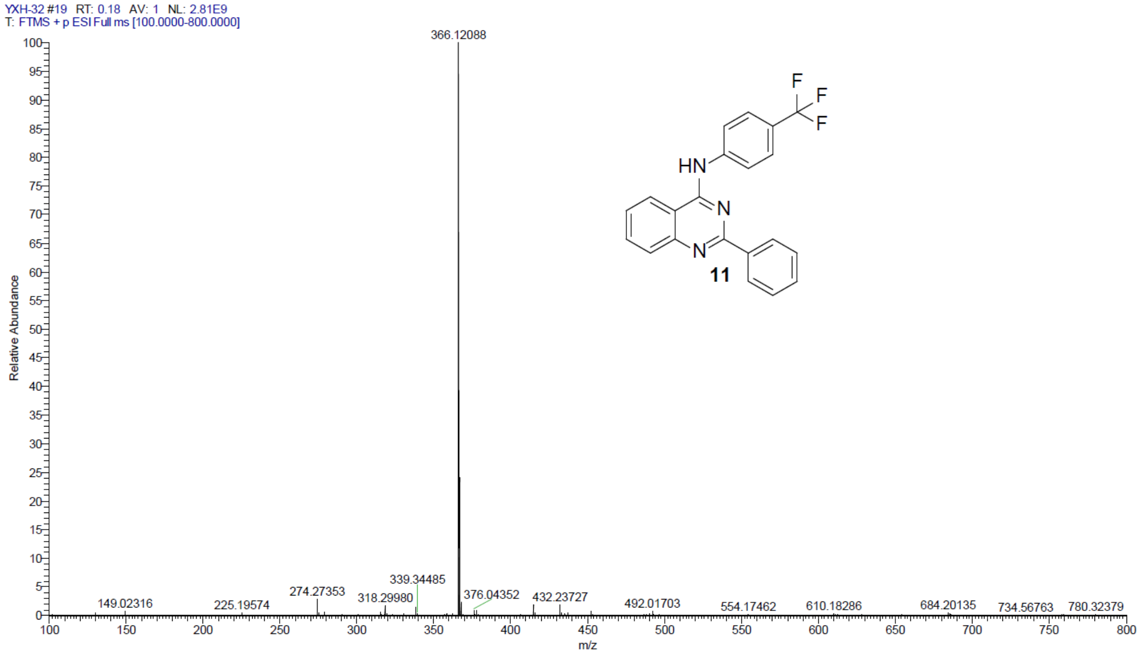

Figure S34 HRMS of 11

HRMS (ESI): m/z calcd for $C_{21}H_{18}N_3$ $(M+H)^+$, 312.1501; found, 312.1491.

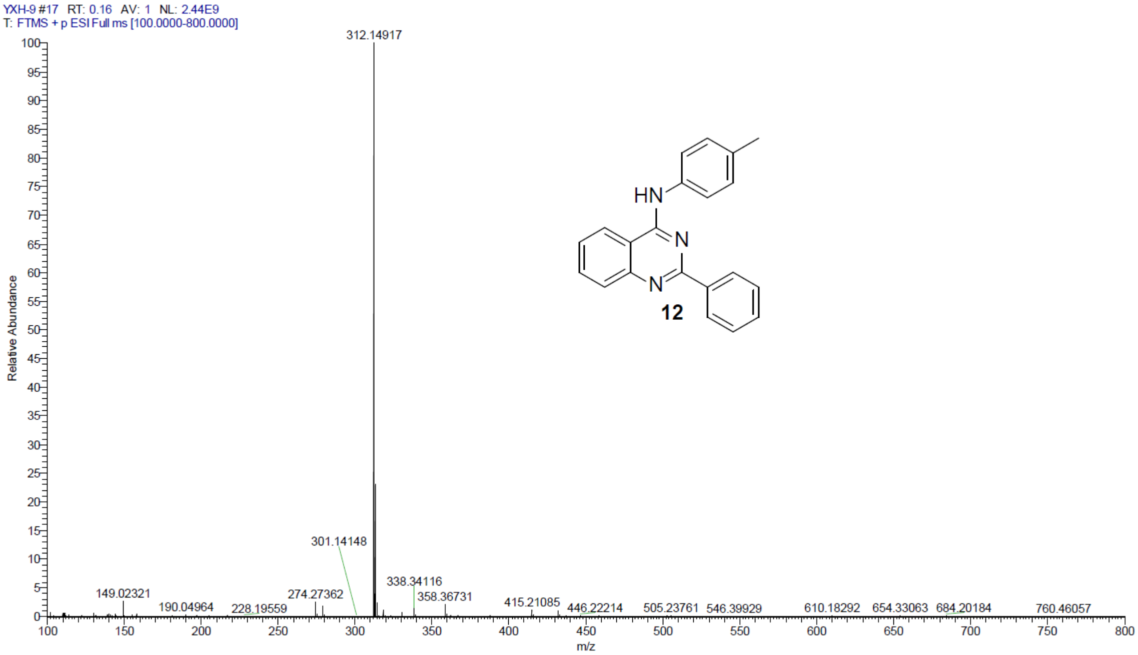

Figure S35 HRMS of 12

HRMS (ESI): m/z calcd for $C_{21}H_{18}N_3O$ $(M+H)^+$, 328.1450; found, 328.1440.

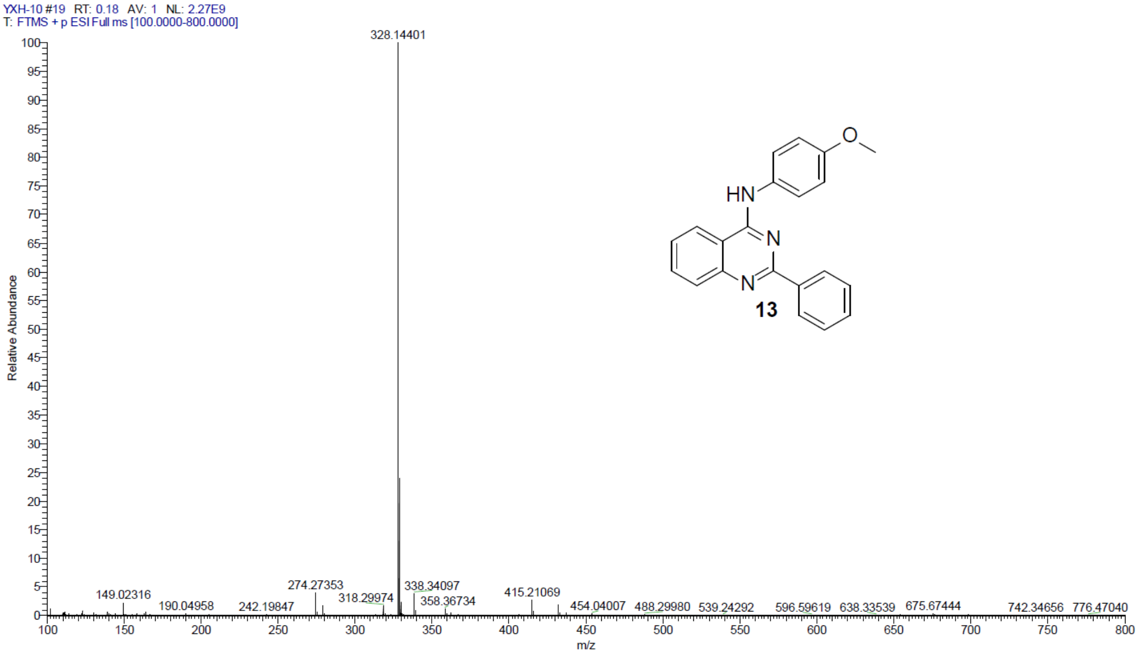

Figure S36 HRMS of 13

HRMS (ESI): m/z calcd for $C_{23}H_{22}N_3O_3$ $(M+H)^+$, 388.1661; found, 388.1650.

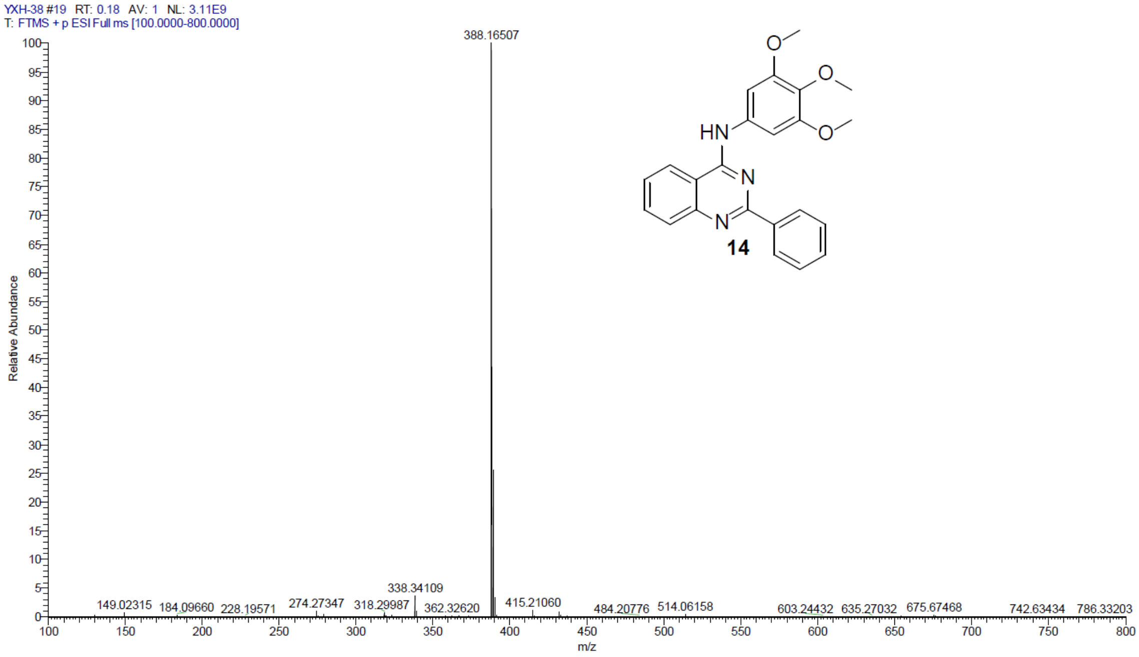

Figure S37 HRMS of 14

HRMS (ESI): m/z calcd for $C_{24}H_{18}N_3$ $(M+H)^+$, 348.1501; found, 348.1492.

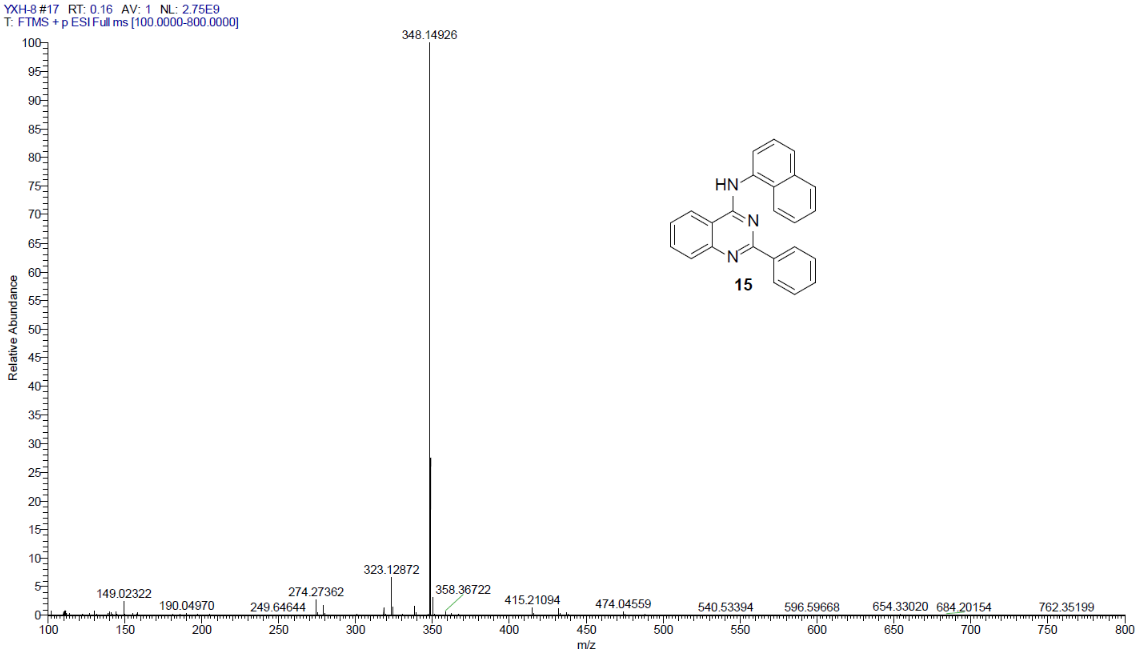

Figure S38 HRMS of 15

HRMS (ESI): m/z calcd for $C_{29}H_{21}FN_3O$ $(M+H)^+$, 446.1669; found, 446.1658.

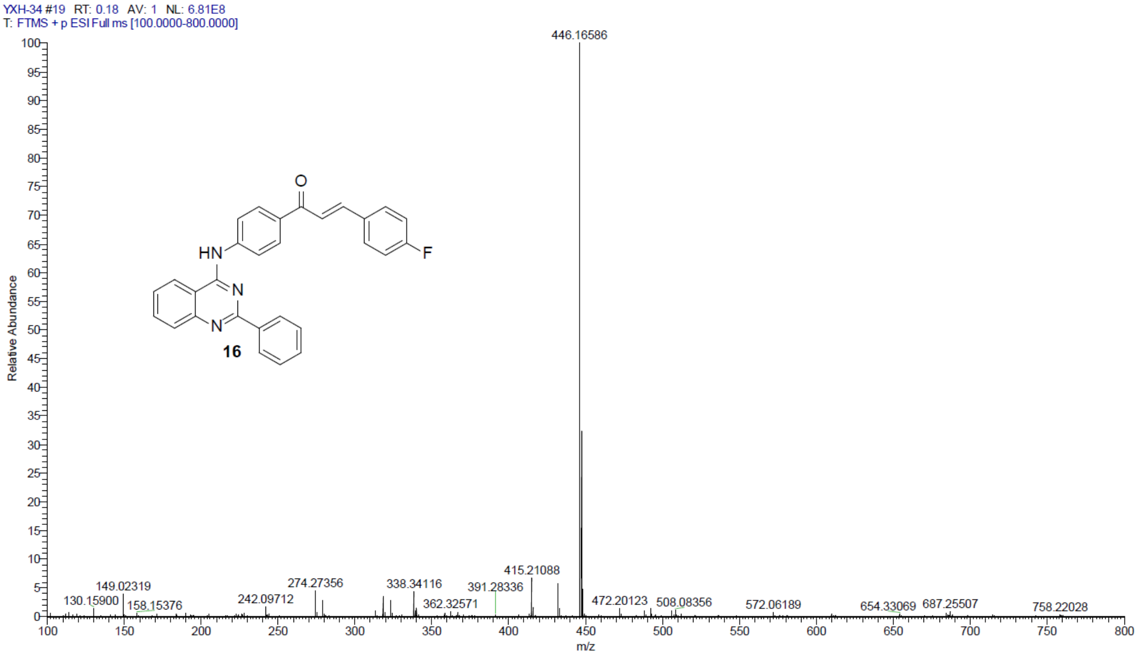

Figure S39 HRMS of 16

HRMS (ESI): m/z calcd for $C_{29}H_{21}ClN_3O$ $(M+H)^+$, 462.1373; found, 462.1365.

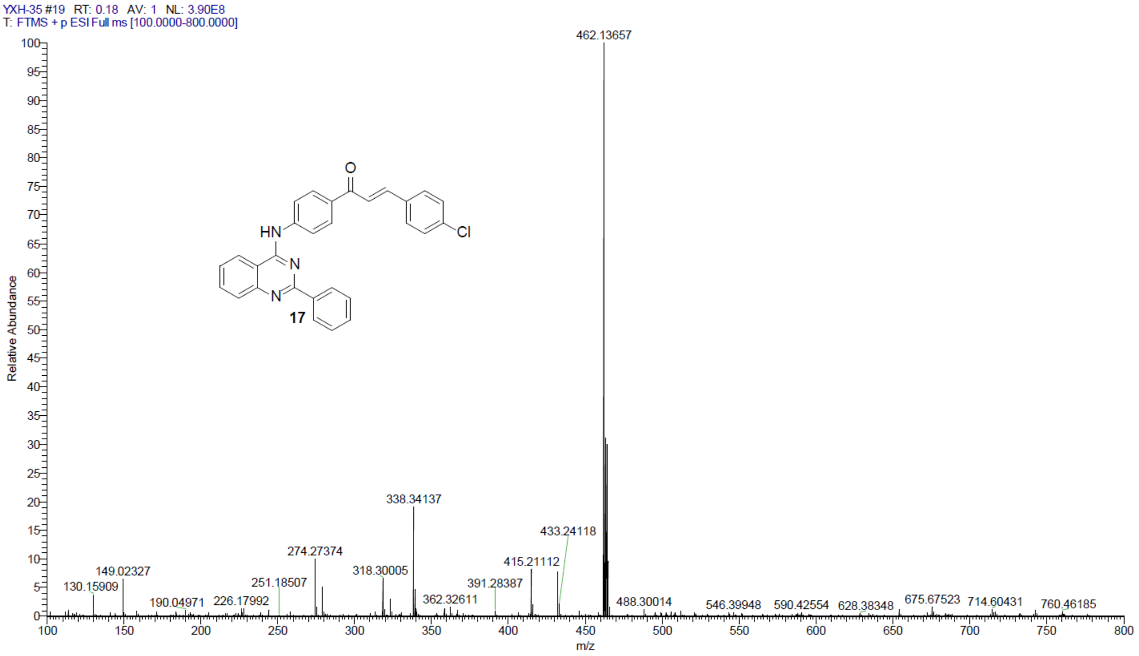

Figure S40 HRMS of 17

HRMS (ESI): m/z calcd for $C_{29}H_{21}BrN_3O$ $(M+H)^+$, 506.0868; found, 506.0860.

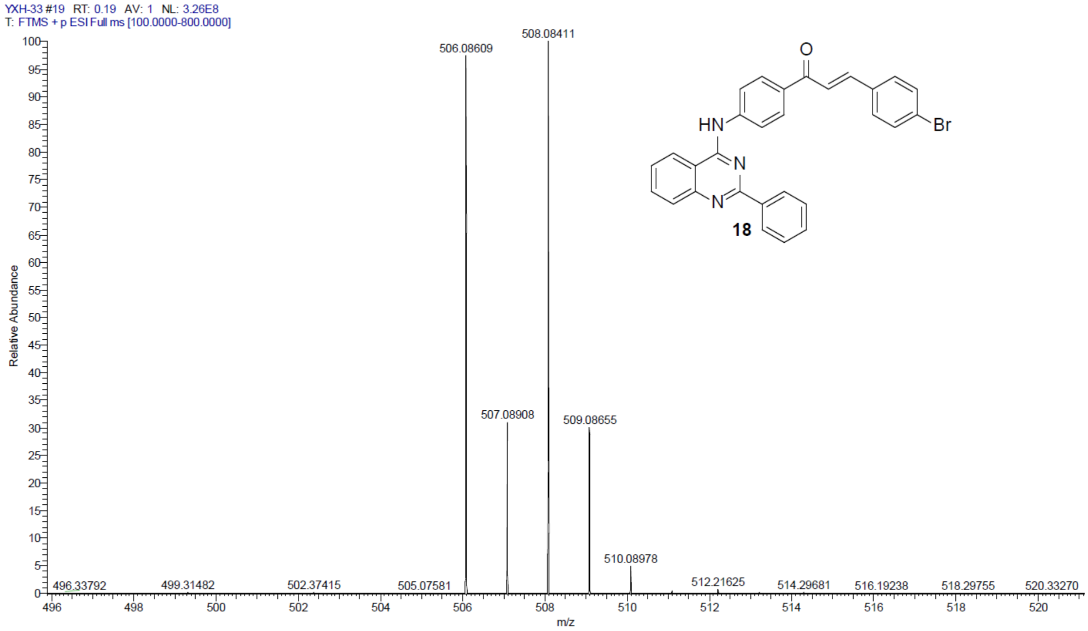

Figure S41 HRMS of 18

HRMS (ESI): m/z calcd for $C_{33}H_{24}N_3O$ $(M+H)^+$, 478.1919; found, 478.1910.

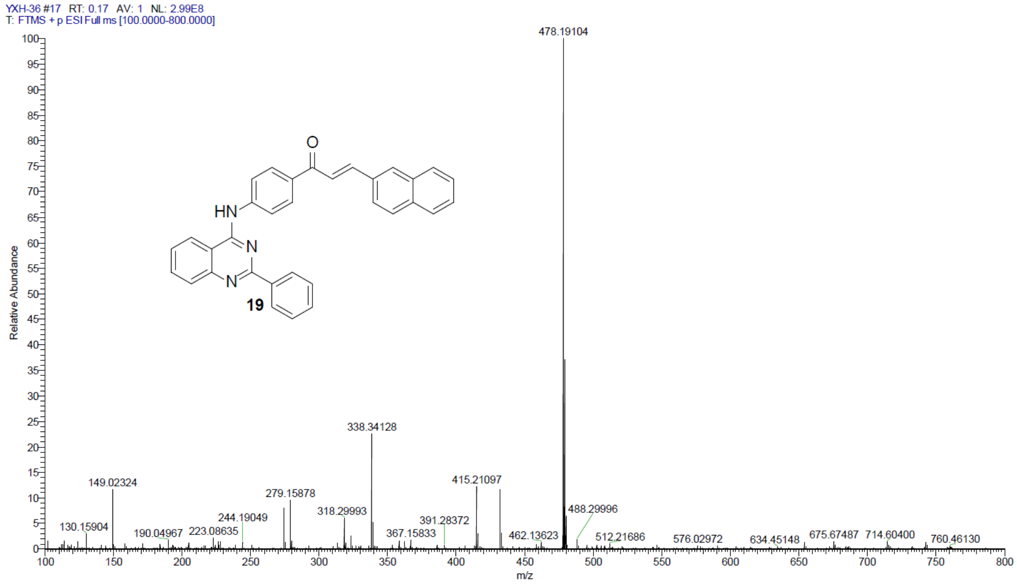

Figure S42 HRMS of 19
